# A phase I trial evaluating the safety, tolerability, pharmacokinetics and pharmacodynamics of intravenously administered low-anticoagulant heparin (M6229) in critically ill sepsis patients

**DOI:** 10.1186/s40635-025-00790-4

**Published:** 2025-08-18

**Authors:** Niels van Mourik, Rombout B. E. van Amstel, Marleen A. Slim, Lonneke A. van Vught, Tom van der Poll, Joram Huckriede, Femke de Vries, Sjef J. de Kimpe, Raf Crabbé, Simone J. M. van Leeuwen, Peter F. Ekhart, Chris P. M. Reutelingsperger, Gerry A. F. Nicolaes, Alexander P. J. Vlaar, Marcella C. A. Müller

**Affiliations:** 1 Department of Intensive Care Medicine, Amsterdam University Medical Center, University of Amsterdam, Meibergdreef 9, 1105AZ Amsterdam, The Netherlands; 2 Center for Experimental and Molecular Medicine, Amsterdam University Medical Center, University of Amsterdam, Amsterdam, The Netherlands; 3 Department of Internal Medicine, Division of Infectious Diseases, Amsterdam University Medical Center, University of Amsterdam, Amsterdam, The Netherlands; 4 Department of Biochemistry, Cardiovascular Research Institute (CARIM), Maastricht University, Maastricht, The Netherlands; 5 Matisse Pharmaceuticals B.V, Geleen, The Netherlands

**Keywords:** Sepsis, Septic shock, M6229, Histones, Neutralization, Phase I, Heparin, Safety, Pharmacokinetics

## Abstract

**Background:**

Histones released in response to cellular injury are important mediators of organ failure and death in sepsis. Preclinical studies demonstrate that neutralization of histones in sepsis is associated with improved outcome. M6229 is a low-anticoagulant heparin able to neutralize histones. We aimed to evaluate the safety, tolerability, pharmacokinetics and pharmacodynamics of M6229 in critically ill patients with sepsis.

**Methods:**

This was a first-in-human, phase I, monocenter trial in patients with sepsis admitted to the intensive care unit (ICU). Patients received a single 6 h intravenous infusion of M6229. A modified continual reassessment method (mCRM) with escalation overdose control was used for dose-escalation. The model was based on the probability of activated partial thromboplastin time (aPTT) being above 90 s (i.e., dose limiting pharmacologic event, DLPE). Three cohorts were studied (1: 0.15 mg/kg/h; 2: 0.45 mg/kg/h; 3: 0.90 mg/kg/h).

**Results:**

Ten patients were included. The aPTT increased proportionally with increasing dosages of M6229 and decreased rapidly after infusion cessation. One DLPE occurred (aPTT of 100 s). Based on the mCRM model and data safety monitoring board recommendations, the maximum tolerated dose was defined as 0.9 mg/kg/h for a 6 h infusion of M6229. No serious adverse events were related to study drug infusion. An increase in QTc was probably related to infusion in one patient. M6229 showed close to dose-proportional pharmacokinetics. Total histone H3 and H2b plasma levels increased during and/or in the hours after M6229 infusion in all patients. In four out of five patients with plasma samples positive for histone H3, proteolytic cleavage was observed after infusion start. A decrease in sequential organ failure assessment score was observed in the days after infusion in 70% of patients.

**Conclusions:**

M6229 was deemed safe to use in critically ill sepsis patients. Our results suggest intravascular neutralization of histones by M6229. Future clinical studies need to confirm our findings and the efficacy of M6229**.**

**Supplementary Information:**

The online version contains supplementary material available at 10.1186/s40635-025-00790-4.

## Background

Histones are major protein components of chromatin in eukaryotic cell nuclei, involved in gene regulation [1]. Extracellular histones are released upon cellular injury and play a pivotal role in the pathophysiology of sepsis. Once in the extracellular space, histones may act as damage-associated molecular patterns. They interact with Toll-like receptors, complement, and phospholipids; damage host endothelial and epithelial cells, platelets, and red blood cells; and induce inflammation and coagulation [2–4]. Subsequently, damaged cells release histones from their nucleus. This results in a self-enforcing feedback cascade, accelerating tissue damage and additional release of histones, ultimately leading to organ failure and death. Indeed, evidence from preclinical studies indicates that neutralization of extracellular histones confers a strong survival benefit in experimental sepsis [5, 6]. In line with this, in patients with sepsis and other types of critical illness, elevated circulating histone levels correlate with increased mortality and adverse outcome [3, 7–12]. To date, no specific drugs are registered for the neutralization of histones in critical illness.

Histones are highly cationic proteins which can be neutralized by polyanions, such as unfractionated heparin (UFH). Unfractionated heparin has been in clinical use for many decades. In animal models of sepsis, low anticoagulant heparin treatment increases survival independent of its anticoagulant activity [13]. In patients with sepsis, UFH treatment was associated with lower plasma histone levels compared to those untreated [14]. Several meta-analyses of clinical trials with UFH or low molecular weight heparins (LMWH) in sepsis showed a significant reduction in 28-day mortality [15, 16]. Nevertheless, it is known that patients with sepsis are at increased risk for bleeding complications due to a dysregulated hemostatic balance [17, 18]. As such, administration of anticoagulant heparins is not desirable and limited to low dosages, hereby limiting the histone neutralizing effect.

M6229 is a low anticoagulant fraction isolated from compendial UFH. M6229 lacks the specific penta-saccharide sequence that is required for the antithrombin-activating properties of UFH [19]. M6229 has shown to neutralize histones in vitro and to improve survival in animal models of sepsis in vivo [13, 20]. Being a low-anticoagulant fraction of UFH, M6229 is characterized as a heparin with a 2-orders of magnitude lower inhibition of factor Xa [13]. Therefore, in theory, M6229 can be administered to human subjects at higher dose levels compared to UFH to exert its histone-neutralizing effect, without exposing the patient to an unacceptable bleeding risk.

The objective of this study was to investigate M6229 as an add-on therapy for patients with sepsis, by conducting an exploratory phase I study. This first-in-human clinical study evaluated the safety, tolerability, pharmacokinetics (PK) and pharmacodynamics (PD) of intravenously administered M6229 in critically ill patients with sepsis.

## Methods

This first-in-human study for M6229 was conducted in critically ill patients with sepsis at one study center. It was a single ascending intravenous dose study. The trial was conducted in accordance with International Conference on Harmonization Good Clinical Practice guidelines, the principles of the Declaration of Helsinki, the Medical Research Involving Human Subjects Act and other (institutional) guidelines, regulations and acts. It was approved by the Dutch Central Committee on Research Involving Human Subjects (CCMO; registration number: NL77116.000.21; date of approval: July 14, 2021) and the Dutch Ministry of Health, Welfare and Sport as competent authority. The trial was registered with www.clinicaltrials.gov (NCT05208112; “HistoSeps”). This study was reported in accordance with the Consolidated Standards of Reporting Trials (CONSORT) Dose-finding Extension.

### Patient population

Eligible participants were adult patients (≥ 18 years) admitted to the intensive care unit (ICU) of Amsterdam University Medical Center (UMC), Amsterdam, The Netherlands, diagnosed with sepsis according to Sepsis-3 criteria [21]. Inclusion criteria required patients to be admitted or diagnosed with sepsis in the ICU within 72 h. M6229 administration had to occur within 84 h of ICU admission or sepsis diagnosis. Patients with a high bleeding risk were excluded (e.g., using therapeutic anticoagulants or patients with severe thrombocytopenia). Informed consent was required from patients or their legal representatives. For a full overview of the in- and exclusion criteria, see Appendix I (Supplementary Material).

### Protocol amendments

After the CCMO approved a protocol amendment on February 8, 2022, the in- and exclusion criteria were adjusted to permit the inclusion of patients with coronavirus disease 2019 (COVID-19) and/or severe renal insufficiency (eGFR < 30 mL/min; dialysis dependency). This revision was made by the recognition that the presence of COVID-19 in ICUs was likely to persist for an extended period, and COVID-19 patients in the ICU generally meet the Sepsis-3 criteria for sepsis. Additionally, since UFH is renally excreted in its inactive form, and renal insufficiency does not preclude the use of UFH (thereby M6229), including renal insufficiency was deemed appropriate. Furthermore, another amendment to the protocol, approved on October 20, 2022, adjusted the inclusion criteria to permit the inclusion of patients with ICU-acquired sepsis, in addition to those initially admitted with sepsis.

### Dose escalation

**Fig. 1:**
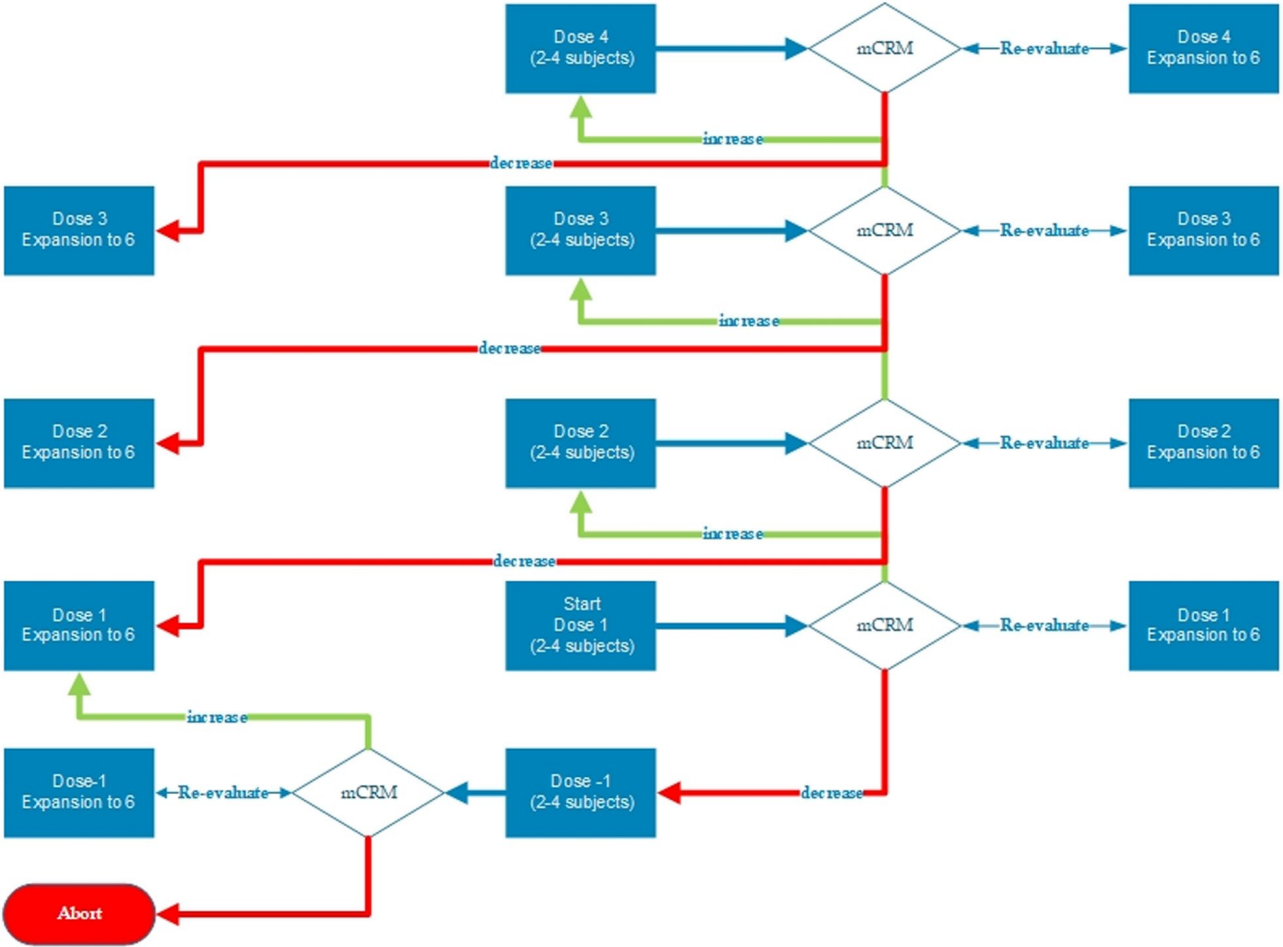
Dose escalation scheme using the modified continual reassessment method

Dose escalation was based on a Bayesian modified continual reassessment method (mCRM) including escalation with overdose control (EWOC). The mCRM was based on modeling the probability of activated partial thromboplastin time (aPTT) being above 90 s (> 3 times the upper limit of normal (ULN)), i.e., a dose limiting pharmacologic event (DLPE). Frequent and sequential measurement of the aPTT allowed for rapid detection of any excessive anticoagulant effect in individual subjects. The target probability of experiencing a DLPE ranged between 1/6 to 1/3. The use of the EWOC principle limits the risk of exposing participants in the next cohort to an intolerable dose by ensuring that the posterior probability of experiencing a DLPE exceeding 1/3 at any dose was capped at 0.45. The DLPE period started at the initiation of M6229 infusion and ended 24 h after the initiation of M6229 infusion. Subjects who received at least 80% of the planned M6229 dose, had an aPTT level measured at the end of the infusion and completed infusion, or discontinued due to a DLPE during the observation period were considered as DLPE evaluable. Dose escalation would continue until the maximum tolerated dose was defined. The number of DLPE evaluable subjects could not exceed 26 if the data safety monitoring board followed all mCRM including EWOC dose recommendations. A schematic flowchart of the study design is shown in Fig. 1.

### Data safety monitoring board (DSMB)

The DSMB was consulted before every dose-escalation step and consisted of an independent statistician, intensivist and hospital pharmacist. The DSMB had access to all data available. When a dose cohort was closed, the DSMB was consulted either to accept or overrule the mCRM recommendation. During the DSMB evaluation period, new patients could not be included in the study.

### Intervention

Patients received a single 6 h infusion of M6229. The starting dose of M6229 was 0.15 mg/kg/h, five times higher than the heparin content of a therapeutic dose of UFH. Next, the dose was escalated to 0.45 mg/kg/h and finally to 0.9 mg/kg/h, as recommended by the mCRM modelling and endorsed by the DSMB. When a patient’s weight was > 100 kg, the patient specific dosing was calculated for 100 kg.

### Aims

The primary aim was to assess the safety, tolerability, pharmacokinetics and pharmacodynamics of M6229. Secondarily, the effects of M6229 on markers for severity of illness were explored.

### Data collection

Safety data were collected through physical exams (e.g., bleeding signs), vital signs (heart rate, blood pressure, respiratory rate, pulse oximetry, temperature), adverse events (per Common Terminology Criteria for Adverse Events 5.0), blood tests (hematology, biochemistry, coagulation), and electrocardiography. The Fridericia formula was used to determine corrected QT time (QTcF, i.e., QTc = QT interval/(RR interval)^1/3^). PK data included plasma parameters (Tmax, Cmax, AUC0-6 h, AUClast, AUCinf, t1/2, CL, Vd) and urine parameters (Ae_x-y_, Ae_total_, D_urine,x-y_, D_urine,total_ and CL_R_) for M6229. PD measures involved Histone H3, H2b levels and H3 cleavage in citrated plasma. Moreover, daily Sequential Organ Failure Assessment (SOFA) scores were collected. Patients were monitored for 72 h post-infusion, with PK and histone samples collected at specified intervals up to 72 h after the start of infusion. Sampling ceased upon ICU discharge, with a clinical follow-up 30 days post-infusion. See Appendix II and III (Supplementary Materials) for the assessment schedule and adverse event definitions, respectively.

### Pharmacokinetics

Ardena Bioanalysis B.V., in Assen, The Netherlands performed the bioanalysis. For this purpose, a quantitative fluorescence assay was validated. Plasma or urine samples containing M6229 were pipetted into a 96 wells plate, followed by addition of Heparin Red reagent (Redprobes, Münster, Germany) in Enhancer Solution. Fluorescence of the Heparin Red fluorophore was quenched by binding of M6229, resulting in an inverse correlation between M6229 concentration and fluorescence intensity. The fluorescence was measured in a plate reader (excitation wavelength 570 nm, emission wavelength 610 nm). Specificity of this method for M6229 was verified and was not changed in the presence of histones.

The PK analysis was performed by DGr Pharma in Oudenbosch, The Netherlands. PK analysis was performed using the computer program Phoenix^®^ WinNonlin^®^ (version 8.1; Copyright ^©^1998–2018, Certara L.P., USA). Non-compartmental analysis (model type: plasma (200–202), dose type: IV infusion) was applied for the PK analysis of the plasma and urine PK parameters.

### Pharmacodynamics

H3 Western Blot analysis was performed as described previously [10]. This method allowed to observe any proteolytic activity exerted on histone H3 during treatment. Specificity for histone H3 was not affected by M6229. Moreover, a quantitative analysis of histones H2b and H3 was performed by Epidisease, Valencia, Spain, employing liquid chromatography-mass spectrometry (LC–MS) after trypsin digestion. For this analysis, stable isotope-labeled peptides specific to histones H2b and H3 were used [22]. This approach enabled the simultaneous quantification of both histones in a single analytical run. Notably, the method maintained its specificity in the presence of the compound M6229.

### Statistical analysis

With the exception of the mCRM with EWOC that was run, no formal statistical analyses were conducted. Instead, descriptive analyses were performed to provide insights into the data trends and distributions. Our descriptive analysis included measures as mean, median, range and standard deviation, alongside graphical representations. All analyses were performed using SAS 9.4 (or higher) or R 4.0 (or higher).

## Results

### Screening and inclusion

**Fig. 2:**
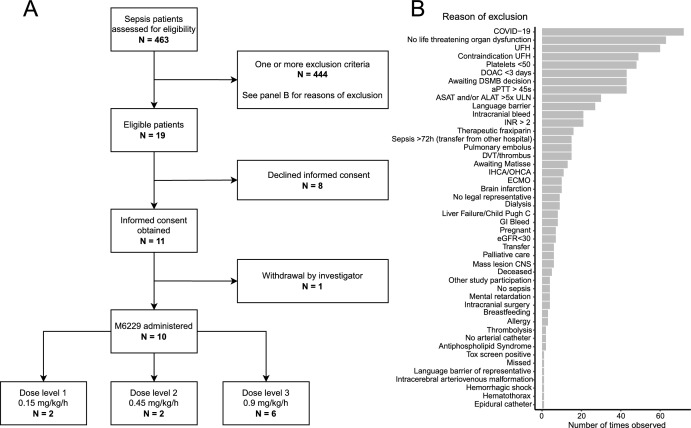
Flowchart of the inclusion process (**A**) and reasons for exclusion (**B**). Patients with COVID-19 and renal failure were excluded up to February 8, 2022, thereafter an amendment to the protocol was approved and these patients were considered eligible. The total occurrence of exclusion criteria was counted; i.e., if multiple exclusion criteria applied to one patient, multiple bars were incremented

Of 463 screened sepsis patients, 444 patients were excluded, leaving 19 eligible. Figure 2 shows an overview of the in- and exclusion process. Informed consent was obtained from 11 patients. The first patient was included on April 5, 2022, the last follow-up was on September 28, 2023. One participant (AMC001) was withdrawn by the investigators prior to M6229 infusion, due to the initiation of extra-corporeal membrane oxygenation and hereby the necessity to administer UFH. Ultimately, 10 patients received M6229. Two patients were included in dose level 1 (0.15 mg/kg/h); two patients in dose level 2 (0.45 mg/kg/h), and 6 patients in dose level 3 (0.9 mg/kg/h).

### Study population

All patients were DLPE evaluable. For a detailed breakdown of prescribed and actual dosages administered and DLPE evaluability, refer to Table 1.

**Table 1:** Study drug dosing

Dose levels	Patient	Weight (kg)	Dose prescribed (mg)	Dose administered (mg)	Percentage of prescribed dose administered (%)	DLPE evaluable
Dose level 1 (0.15 mg/kg/h)	*AMC002*	72.5	65.25	66.0	101.0	Yes
	*AMC003*	80.0	72.00	72.0	100.0	Yes
Dose level 2 (0.45 mg/kg/h)	*AMC004*	59.0	159.30	135.0	84.7	Yes
	*AMC005*	121.0*	270.30	230.0	85.1	Yes
Dose level 3 (0.9 mg/kg/h)	*AMC006*	92.5	499.50	420.0	84.0	Yes
	*AMC007*	72.4	390.96	330.0	84.4	Yes
	*AMC008*	82.4	444.96	450.0	101.0	Yes
	*AMC009*	72.0	388.80	388.8	100.0	Yes
	*AMC010*	78.5	423.90	430.0	101.4	Yes
	*AMC011*	72.5	391.50	394.0	100.6	Yes

Subjects who have received at least 80% of the planned M6229 dose, have an aPTT level measured at the end of the infusion and have completed or discontinued due to a DLPE during the observation period were DLPE evaluable.*As the patient its weight was > 100 kg, the patient specific dosing was calculated for 100 kg

### Patient characteristics and outcomes

In our cohort of 10 patients, 40% were female (Table 2). The patients’ ages ranged from 50 to 75 years, with a median age of 67 years. The SOFA score on the day of infusion ranged from 3 to 10 with a median of 8. Pneumosepsis was diagnosed in most participants (n = 6, 60%), other sources of sepsis included the gastrointestinal tract (n = 1, 10%), central nervous system (n = 1, 10%), skin (n = 1, 10%), and bloodstream (n = 1, 10%). Acute respiratory distress syndrome (ARDS) during ICU admission was observed in 6 patients (60%); all patients suffered from severe ARDS according to the Berlin criteria [23]. Moreover, 4 patients (40%) were in need of renal replacement therapy during ICU admission. The mortality rate in the ICU was 30% until the day 30 last follow up of this study; there were no subsequent deaths after patients were discharged to the ward.

**Table 2:** Patient characteristics

	Dose level 1		Dose level 2		Dose level 3					
	*AMC002*	*AMC003*	*AMC004*	*AMC005*	*AMC006*	*AMC007*	*AMC008*	*AMC009*	*AMC010*	*AMC011*
Demographics										
Age (years)	71	67	64	70	55	67	75	68	50	64
Sex	Female	Male	Female	Male	Female	Male	Female	Male	Male	Male
Weight (kg)	72.5	80.0	59.0	121.0	92.5	72.4	82.4	72.0	78.5	72.5
BMI (kg/m^2^)	23.7	22.2	19.9	35.4	30.9	23.9	27.2	22.2	21.8	24.5
Admission data										
Admission type	Medical	Medical	Medical	Medical	Medical	Medical	Surgical	Surgical	Surgical	Medical
Hospital admission diagnosis	COVID-19	Encephalitis	Sepsis	Sepsis	COVID-19	Pneumonia and spondylodiscitis	Infected neuropathic ulcus requiring amputation	Supracoronary hemi-arch replacement	Type B dissection complicated by aortitis	Sepsis
Sepsis source	Respiratory tract	Central nervous system	Respiratory tract	Abdominal	Respiratory tract	Respiratory tract; Bloodstream	Skin	Respiratory tract	Bloodstream	Respiratory tract; Bloodstream
Pathogen isolated	SARS-CoV 2	Japanese Encephalitis Virus	Rhinovirus	NA	SARS-CoV 2	S. aureus	S. pyogenes	K. pneumoniae; E. cloacae	S. aureus	S. aureus
Severity of illness										
SOFA score on infusion day	8	8	9	9	4	8	10	8	6	10
APACHE IV score	67	57	111	75	68	68	82	45	48	158
ARDS	Yes, severe	No	Yes, severe	No	Yes, severe	Yes, severe	No	Yes, severe	No	Yes, severe
RRT	No	Yes	No	No	Yes	Yes	No	No	No	Yes
Clinical outcome										
ICU mortality	No	Yes	No	No	Yes	No	No	No	No	Yes
30 day mortality	No	Yes	No	No	Yes	No	No	No	No	Yes
Cause of death	Ongoing lung disease, palliative trajectory	Unknown (transferred to other hospital)	NA	NA	New sepsis episode, multi-organ failure	NA	NA	NA	NA	Multi-organ failure

BMI: denotes Body Mass Index; SOFA: Sequential Organ Failure Assessment; APACHE: Acute Physiologic and Chronic Health Evaluaton; ARDS: Acute Respiratory Distress Syndrome (as per the Berlin criteria [23]); RRT: renal replacement therapy; ICU: intensive care unit; NA: not applicable or not available

### Safety and tolerability

One patient experienced a DLPE, this occurred at dose level 3 (0.9 mg/kg/h). Due to this event and previously observed aPTTs, based on the mCRM and DSMB recommendations, it was decided to not further increase the dosage to 1.25 mg/kg/h; but to expand the third cohort (0.9 mg/kg/h) to 6 patients. As such, a dose level of 0.9 mg/kg/h was found to be the maximum tolerated dose (MTD). The aPTTs pre-dose and at the end of infusion are presented in Table 3.

**Table 3:** aPTT pre-dose and at end of infusion per patient

Dose levels	Patient	aPTT (s) pre-dose (T0)	aPTT (s) end of infusion (T6)	Absolute change in aPTT (s)	Relative change (%)
Dose level 1	*AMC002*	< 20	25	5	25.0
	*AMC003*	30	37	7	23.3
Dose level 2	*AMC004*	34	49	15	44.1
	*AMC005*	27	40	13	48.2
Dose level 3	*AMC006*	< 20	73	53	265.0
	*AMC007*	33	44	11	33.3
	*AMC008*	< 20	61	41	205.0
	*AMC009*	23	70	47	204.4
	*AMC010*	27	100	73	270.4
	*AMC011*	< 20	69	49	245.0

**Fig. 3:**
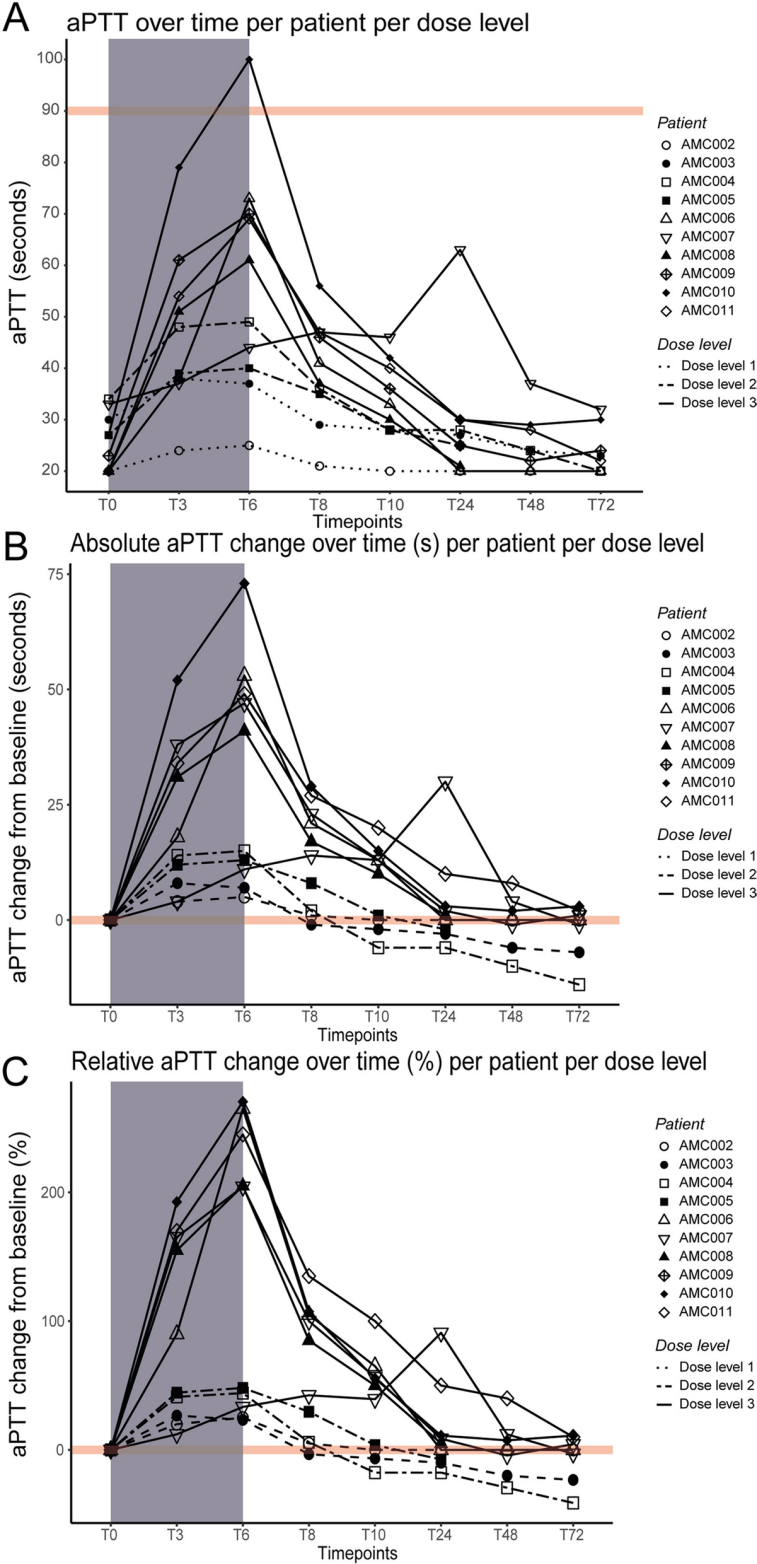
aPTT over time per patient per dose level. The infusion period is displayed in grey. Patient distinction can be made by point shape. Dose level distinction can be made by line type. **A** Displays the aPTT profile over time, with the red line marking the DLPE threshold at an aPTT of 90 s. **B** Shows the absolute change in aPTT, measuring the difference in seconds from baseline. **C** Depicts the relative change in aPTT, calculated as a percentage compared to the aPTT at baseline. aPTTs below the lower limit of detection (< 20) were defined as 20

Figure 3 depicts the aPTT changes over time; before, during and after infusion (panel A), including both absolute (panel B) and relative changes (panel C) compared to baseline. There was a dose-dependent increase in aPTT, with absolute changes in aPTT (T0 vs. T6) ranging between 5–7 s for dose level 1, 13–15 s for dose level 2, and 11–73 s for dose level 3. The increase occurred rapidly after the start of infusion, peaking at the end of infusion. Values returned to baseline quickly after drug cessation. The largest increase in aPTT was found between pre-dose (T0) and 3 h (T3), except for AMC006, demonstrating a continuous increase up until T6 (end of infusion). In AMC006, an approximately 2 h pause in infusion occurred, as intravenous access was required for intubation. T3 was collected approximately 1.5 h after infusion pause and 30 min before restart of the infusion; T6 was collected at the end of infusion.

A decline in aPTT post-M6229 therapy was observed in all subjects, except for AMC007. AMC007 demonstrated a smaller increase in aPTT compared to other subjects (33.3% increase between start and end of infusion). In addition, only for AMC007 an increase in aPTT was observed after end of infusion, with a notable increase in aPTT between 10 (T10) and 24 h (T24) after start of infusion. This increase between T10 and T24 can be explained due to the protocol-driven initiation of continuous veno-venous hemodialysis with continuous administration of a low dose of UFH.

No changes in other laboratory tests or vital parameters with regards to safety and tolerability were deemed probably or certainly related to infusion. No serious adverse events were deemed related to M6229 infusion (see appendix IV).

An increase in QTcF was observed during study drug infusion in one patient (AMC010: T0, 461 ms; T6, 504 ms; T24, 472 ms). This was considered clinically relevant due to the significant increase, exceeding 500 ms. The event was assessed as probably related to M6229, given the temporal association with drug administration, the absence of alternative explanations, and resolution following discontinuation of the study drug.

Refer to Appendix IV, V, VI, VII, VIII, IX (Supplementary Materials), for an overview of the adverse events, vital signs, hematology, coagulation, biochemistry and electrocardiography variables, respectively.

### Pharmacokinetics

**Fig. 4:**
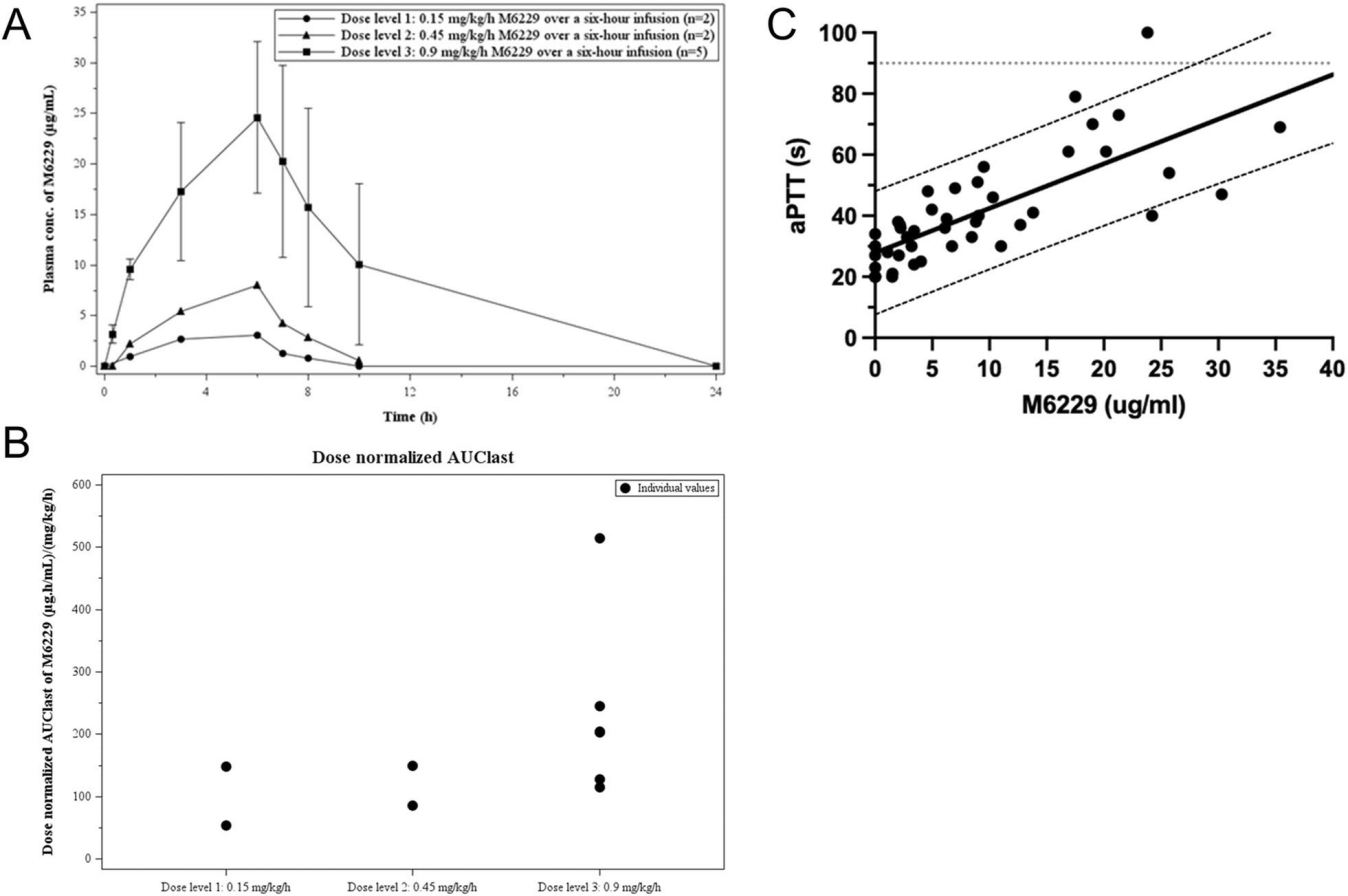
Pharmacokinetics. **A** displays the mean plasma concentration–time profiles of M6229 on a linear scale for 0.15 mg/kg/h M6229 (Dose level 1); 0.45 mg/kg/h M6229 (Dose level 2); 0.9 mg/kg/h M6229 (Dose level 3). Means and SDs are shown. AMC007 is excluded from this graph. **B** displays a scatter plot of plasma dose normalized pharmacokinetic parameters of M6229 on a categorical scale for all patients. **C** shows the correlation between plasma levels of M6229 and aPTT over the first 24 h period for all patients except AMC007, including a 90% prediction interval. The dotted line marks the DLPE threshold at 90 s

Mean plasma-concentration time profiles of M6229 are shown in Fig. 4A. The PK parameters of M6229 in critically ill sepsis patients are summarized in Table 4. Following UFH treatment after T10 in patient AMC007, M6229 concentration levels could not be reliably measured, leading to its exclusion from the pharmacokinetic analyses.

**Table 4:** Summarized pharmacokinetic parameters of M6229 after administration of 0.15 mg/kg/h, 0.45 mg/kg/h or 0.90 mg/kg/h over a six-hour infusion

Pharmacokinetics of M6229	Dose level 1: 0.15 mg/kg/h M6229 over a six-hour infusion	Dose level 2: 0.45 mg/kg/h M6229 over a six-hour infusion	Dose level 3: 0.9 mg/kg/h M6229 over a six-hour infusion
mean ± SD, t_max_ and t_last_: median (range)			
n	2^a^^,b^	2^a^	5^c^
C_max_, µg/mL	2.16–4.01	6.99–9.05	23.9 ± 6.65
t_max_, h	6.02–6.17	6.03–6.08	5.98 (5.62–7.85)
AUC_0-6h_, µg.h/mL	8.01–16.8	24.8–32.0	91.8 ± 26.9
AUC_last_, µg.h/mL	8.21–22.8	32.9–47.2	205 ± 149
t_last_, h	6.02–8.08	8.00–9.83	11.13 (9.33–22.65)
AUC_**∞**_, µg.h/mL	25.8	36.7–49.1	160 ± 27.0
t_1/2_, h	1.36	1.19–1.24	3.75 ± 3.44
V_z_, L	5.05	6.31–8.40	8.73 ± 2.54
CL, L/h	2.56	3.68–4.68	2.68 ± 0.435

AMC007 was excluded from this table as this subject received a UFH infusion from 10-h onwards

^a^Individual values are shown

^b^n = 1 for AUC_**∞,**_ t_1/2_, V_z_ and CL

^c^n = 4 for AUC_**∞**_, V_z_ and CL

Cmax was reached at the end of the infusion and rapidly dropped after infusion cessation. In general, PK of M6229 in these patients was predictable, with close to dose-proportional PK over the investigated dose range. This is demonstrated in Fig. 4B, where the dose-normalized exposure expressed as AUC is shown as function of the administered dose level. Moreover, a linear relationship between M6229 plasma levels and aPTT over the first 24 h was observed (Fig. 4C).

Urine pharmacokinetics indicate that renal clearance and excretion of M6229 is very low in patients (< 3% of the administered dose), see Appendix X (Supplementary Material).

### Pharmacodynamics

**Fig. 5:**
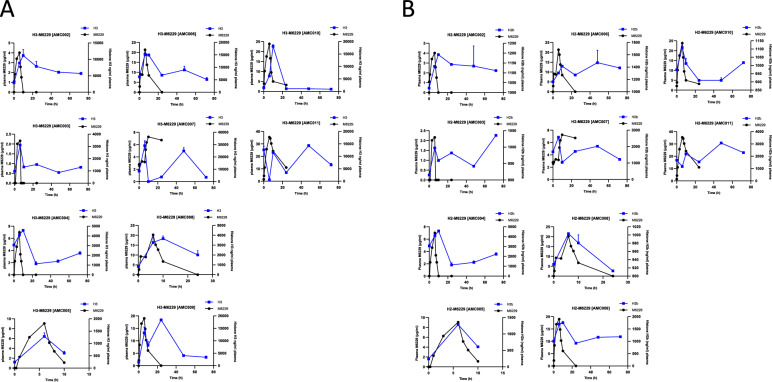
Histone levels over time per patient correlated with M6229 levels. **A** M6229 and histone H3 levels in plasma over time. **B** M6229 and histone H2b levels over time. Plasma levels are found on the y-axis. The x-axis denotes time after infusion start in hours

Histone H3 cleavage was detected in approximately 47% of all longitudinal samples measured. Notably, in the subset of patients where cleavage was observed (n = 5), it was detected in four out of five patients after start of infusion. See Appendix XI (Supplementary Material) for an example on Histone H3 cleavage. Using mass spectrometry, histones H3 and H2b were measurable in all samples. A notable trend was observed in most patients, where H3 plasma levels were increased at the end of infusion and/or T = 10 h, correlating with plasma M6229 levels (Fig. 5A). A similar trend was observed for histone H2b plasma levels, with increases at the end of infusion and 10 h after infusion, and a subsequent decline in most patients (Fig. 5B).

### Effects of M6229 on markers of severity of illness

**Fig. 6:**
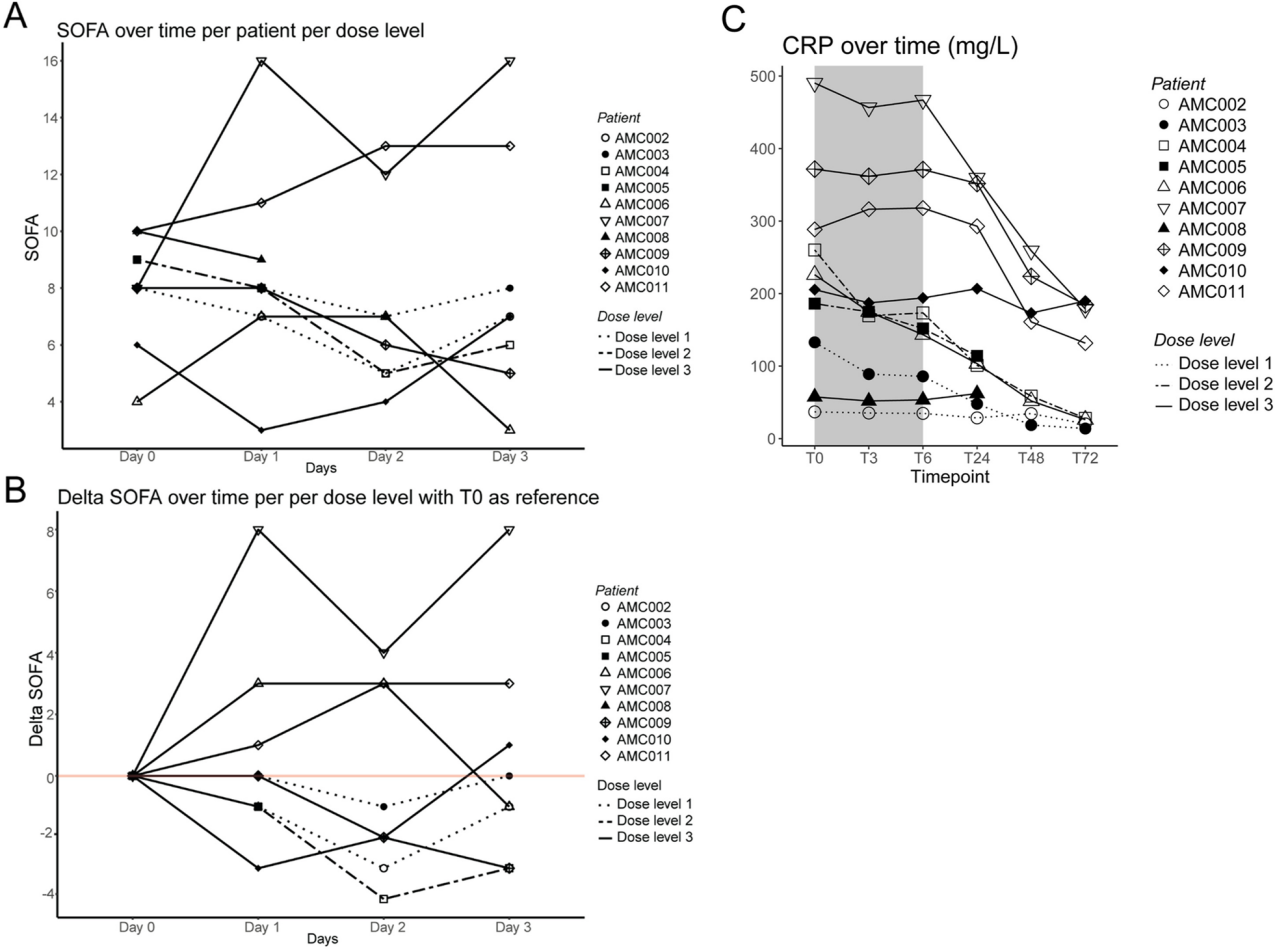
Effects of M6229 on markers of severity of illness. **A** SOFA score over time per patient per dose level. Patient distinction can be made by point shape. Dose level distinction can be made by line type. Day 0 is the SOFA score on infusion day; day 1–3 the days after the day of infusion. **B** Delta SOFA score over time per patient per dose level. Patient distinction can be made by point shape. Dose level distinction can be made by line type. Day 0 is the SOFA score on infusion day; day 1–3 the days after the day of infusion. **C** CRP levels (mg/L) over time per patient per dose level. The infusion period is displayed in grey. Patient distinction can be made by point shape. Dose level distinction can be made by line type

A decrease in SOFA score was found for 70% of the patients following the three days after the infusion day (Fig. 6A and B). For most patients, a decrease was observed in CRP levels following M6229 infusion (Fig. 6C).

## Discussion

In this first-in-human phase I trial, we found that M6229 was safe to use in critically ill patients with sepsis. The maximum tolerated dose was identified as 0.9 mg/kg/h for a single 6 h infusion. One adverse event, an increase in QTc, was considered probably related to study drug infusion. However, given the limited study size, definitive conclusions with regards to this adverse event cannot be drawn.

Treatment of sepsis is moving from a one-size-fits-all approach towards a more personalized approach [24]. This shift underscores the importance of identifying and targeting specific substrates that may offer therapeutic benefits to distinct patient subgroups [24, 25]. Extracellular histones, known for their cytotoxic effects, can be detrimental not only to pathogens but also to host cells, and targeting of histones is deemed a potential strategy to alleviate inflammatory disease and sepsis [26]. Multiple in-vitro and in-vivo studies have shown promising results for different agents that form complexes with histones [13, 27]. Our study is the first to explore modified heparin as a targeted histone-neutralizing agent in a human population, administered to critically ill patients with sepsis. Bellomo et al. recently published a phase-1 study of histone-neutralizing polyanion molecule STC3141 in critically ill patients with sepsis. They showed infusion was feasible and reasonably safe, however, one patient experienced major hemorrhage [28]. Moreover, beyond its histone-neutralizing effects, a previous study demonstrated that UFH also targets Drp1, an important mediator of mitochondrial dysfunction in critical illness, thereby protecting mitochondrial integrity, reducing vascular leakage, and improving sepsis prognosis [29].

M6229 showed close to dose-proportional PK in critically ill patients with sepsis. Moreover, we observed an apparent linear dose–response relationship between M6229 dosage and aPTT values. As M6229, a low-anticoagulant fraction of UFH, exhibits substantially lower factor Xa inhibition, it permits higher dosing in humans without significantly elevating bleeding risk. This is crucial for sepsis patients with concomitant coagulopathy, in whom immunothrombosis plays a pivotal pathophysiological role [30]. In our study, the initial M6229 dose, five times higher than typical therapeutic UFH doses, led to a modest 25% increase in aPTT, well below therapeutic thresholds. Tripling the dose in a second cohort resulted in an approximately 45% aPTT increase, potentially significant for those with already high aPTT levels. The third cohort, receiving six times the initial dose, showed clinically meaningful aPTT elevations within therapeutic ranges (44–100 s). It is important to note that after infusion stop, aPTT levels returned to baseline promptly.

Accepting increased aPTT levels might be justified if it yields substantial patient benefit. Additionally, in patients with sepsis with an indication for anticoagulation, elevated M6229 doses could fulfill a dual role. This aligns with the personalized treatment approach in sepsis; balancing risks and benefits for each patient. However, as this was an exploratory phase I trial with a small sample size, no firm conclusions can be drawn with regards to risk and benefit for certain groups of sepsis patients.

Our findings indicate that following the start of M6229 infusion, a considerable proportion of histone H3 underwent proteolytic cleavage. Prior research has established that the cleavage of histone H3 is associated with diminished harmful effects [10, 31]. Literature has shown that novel variants of activated protein C are able to bind, neutralize and degrade histone H3 [32]. As our results show that progressive H3 cleavage was observed during infusion, we hypothesize that M6229 administration may also be associated with accelerated proteolytic cleavage of H3 and putatively other toxic variants of circulating histones. This may contribute to positive outcome. However, this effect could also result from natural disease recovery or other aspects of standard-of-care treatment. Interestingly, during M6229 infusion, there was an observed increase in total histone plasma concentrations, suggesting a temporal relationship. This differs from previous literature, demonstrating stable extracellular histone plasma concentrations during ICU admission in patients with sepsis up to five days [33]. The observed increase could be caused by the highly anionic nature of M6229, which binds the highly cationic extracellular histones originally bound to endothelial glycocalyx [34]. These histones, partly bound to the endothelial cells, whether or not complexed to damage-associated molecular pattern receptors, may be relocated to the intravascular space and form non-cytotoxic aggregates with M6229. We hypothesize that this observed rise in plasma histone levels observed is unlikely to contribute to cellular toxicity, as it may have formed a complex with M6229. However, our current assays were unable to differentiate between free histones and those bound to M6229. Future studies are planned to determine the proportion of bound versus free histones in this context.

Relatively high concentrations of circulating H2b and H3 were found in our cohort. A previous study found that H2b and H3 levels above 435.61 ng/mL and 300.61 ng/mL respectively were associated with septic shock, whereas levels above 400.44 ng/mL and 258.25 ng/mL were associated with disseminated intravascular coagulation in sepsis patients [22]. The levels found in our study were considerably higher, demonstrating the severity of illness of the patients in our cohort.

A decrease in SOFA score was observed in 70% of patients following M6229 infusion. However, attributing this decrease directly to M6229 is challenging, particularly in the absence of a control group for comparison. This is compounded by other treatment effects next to the natural recovery of the underlying diseases. It is noteworthy that in 4 out of the 7 patients with an initial decrease in SOFA score, there was a subsequent increase in the days after stopping the infusion. This observation suggests a potential benefit from repeated or continuous administration of M6229. However, due to the small sample size and lack of comparative data, these findings should be interpreted very cautiously. In line with these findings, a decrease in CRP was observed following M6229 infusion. This decrease may have been caused by the standard of care treatment and natural course of the sepsis episode, however, could also be caused by the hypothesized anti-inflammatory effects of M6229. No definitive conclusions regarding the efficacy of M6229 can be drawn from this study.

An important limitation of our study is the small sample size (n = 10). An mCRM model was employed, which is designed for efficient dose-finding, allowing for conclusive results with fewer participants. As such, patient exposure to overdosing was minimized. While the small sample size restricts the generalizability of our findings, the results of this phase I trial lay the groundwork for future studies. Moreover, as this study was focused on safety, the eligibility criteria were set relatively strict. As such, a significant number of patients screened for sepsis were not included in the study, limiting generalizability. Future trials should consider adopting less restrictive exclusion criteria, administering M6229 to patients indicated for UFH, and evaluating prolonged infusion strategies..

## Conclusions

M6229 was deemed safe to use in critically ill sepsis patients admitted to the ICU. The maximum tolerated dose was 0.9 mg/kg/h for a 6 h infusion of M6229. No serious adverse events were observed related to study drug infusion. Close to dose-proportional PK was found. M6229 infusion might be associated with cleavage of histones and their extraction into the intravascular space, likely in a non-cytotoxic complex. Future studies need to confirm our findings and the efficacy of M6229.

## Supplementary Information

Supplementary Material 1.

Supplementary Material 2.

Supplementary Material 3.

Supplementary Material 4.

Supplementary Material 5.

Supplementary Material 6.

Supplementary Material 7.

Supplementary Material 8.

Supplementary Material 9.

Supplementary Material 10.

Supplementary Material 11.

## Data Availability

The datasets used and/or analysed during the current study are available from the corresponding author on reasonable request.

## Abbreviations

ARDS: Acute respiratory distress syndrome
aPTT: Activated partial thromboplastin time
APACHE: Acute physiologic and chronic health evaluation
CCMO: Committee on research involving human subjects
COVID-19: Coronavirus disease 2019
DLPE: Dose limiting pharmacologic event
DSMB: Data safety monitoring board
EWOC: Escalation with overdose control
ICU: Intensive care unit
LC–MS: Liquid chromatography-mass spectrometry
LMWH: Low molecular weight heparin
mCRM: Modified continual reassessment method
NA: Not applicable or not available
PD: Pharmacodynamics
PK: Pharmacokinetics
QTc: Corrected QT time
QTcF: Corrected QT time following the Fridericia formula
RRT: Renal replacement therapy
SOFA: Sequential organ failure assessment
UFH: Unfractionated heparin
ULN: Upper limit of normal
UMC: University Medical Center
