# Supplementary material for: A phase I trial evaluating the safety, tolerability, pharmacokinetics and pharmacodynamics of intravenously administered low-anticoagulant heparin (M6229) in critically ill sepsis patients
Source: Intensive Care Med Exp. 2025 Aug 18;13:84. doi: 10.1186/s40635-025-00790-4 (PMC12360993; doi:10.1186/s40635-025-00790-4)
Supplement: Supplementary file 1 — Supplementary Material 1. [file 40635_2025_790_MOESM1_ESM.pdf]

Appendix I – Inclusion and exclusion criteria

Inclusion criteria

Each subject had to satisfy all of the following criteria to be enrolled in the study.

1. Male or female patients aged ≥ 18 year.
2. Signed informed consent by patient or legal representative.
3. Diagnosed with sepsis, defined by the Sepsis-3 criteria as a life-threatening organ dysfunction caused by a dysregulated host response to an infection.

   Organ dysfunction is defined by 1 of the following:

   a. Increase in SOFA score of ≥2.
      i. The baseline SOFA score can be assumed to be zero in patients not known to have pre-existing organ dysfunction.
   b. Acute kidney injury
      i. Defined as eGFR < 15 mL/min.
   c. Acute respiratory distress syndrome
      i. Defined by the Berlin criteria.
   d. The need of mechanical ventilation.
   e. Alteration in mental status.
4. The patients had to be included in the study within 72 hours of ICU admission due to sepsis or within 72 hours after sepsis diagnosis on the ICU. M6229 had to be administered within 84 hours after ICU admission due to sepsis or within 84 hours after sepsis diagnosis on the ICU.

Exclusion criteria

Any subject who met any of the following criteria was excluded from participating in the study:

1. Subject had an advance directive to withhold life-sustaining treatments.
2. Subject was breastfeeding or intended to get pregnant within 30 days of enrolling into the study.
3. Subject was of childbearing potential and had a positive pregnancy test.
   a. A woman was considered to be of childbearing potential under the age of 60 years, unless surgically sterile.
4. Clinical suspicion or confirmation of a viral hemorrhagic shock syndrome including, but not limited to, dengue fever.

5. Bleeding risk:
    a. Clinical:
        i. Active bleeding;
        ii. Head trauma;
        iii. Intracranial surgery or stroke in the past 3 months;
        iv. History of intracerebral arteriovenous malformation, cerebral aneurysm or mass lesions of the central nervous system;
        v. Cerebral haemorrhage;
        vi. History of a bleeding diatheses;
        vii. Gastrointestinal bleeding in the past 6 weeks;
        viii. Presence of an epidural or spinal catheter;
        ix. Contraindication for IV therapeutic UFH.
    b. Laboratory:
        i. Platelet count $<50 \times 10^9$/L;
        ii. INR >2.0;
        iii. Baseline aPTT ≥45 seconds prior to enrolment, 1.5x upper limit of normal (ULN).
6. Use of any of the following treatments:
    a. UFH to treat a thrombotic event within 12 hours before infusion;
    b. LMWH within 24 hours before infusion;
    c. Warfarin (if used within 7 days before study entry AND if the INR exceeds 2.0 at enrolment);
    d. Direct oral anticoagulant (DOAC) use 3 days prior to enrollment.
    e. Thrombolytic therapy within 3 previous days;
    f. Use of IIb/IIIa inhibitors within the previous 7 days.
7. Confirmed antiphospholipid syndrome.
8. Known allergy to fish.
9. Cardiopulmonary resuscitation in the previous 7 days.
10. Liver failure defined as Child-Pugh Score Class C.
11. Abnormal liver function (ASAT and/or ALAT > 5 times upper limit of normal (ULN)).
12. Extracorporeal membrane oxygenation (ECMO) support dependent.
13. Pulmonary embolism or clinical suspicion of deep venous thrombosis (DVT).
14. Life expectancy of less than 24 hours.
15. Treating physician refusal.

16. Known adverse reaction to UFH, including heparin induced thrombocytopenia (HIT).
17. Participation in any other investigational drug study or other interventional study with interfering endpoints.
18. Any other clinical condition which, in the opinion of the investigator, would not allow safe completion of the protocol.
