# Supplementary material for: A phase I trial evaluating the safety, tolerability, pharmacokinetics and pharmacodynamics of intravenously administered low-anticoagulant heparin (M6229) in critically ill sepsis patients
Source: Intensive Care Med Exp. 2025 Aug 18;13:84. doi: 10.1186/s40635-025-00790-4 (PMC12360993; doi:10.1186/s40635-025-00790-4)
Supplement: Supplementary file 2 — Supplementary Material 2. [file 40635_2025_790_MOESM2_ESM.pdf]

Appendix II – Schedule of Assessments

Phase	**Screening / Baseline**	**Pre-dose**	**Continuous IV infusion with M6229**					**Post IV infusion**						**Long term follow-up (Day 30) or Early Withdrawal**
			**Time after Start of Infusion**					**Time after Start of Infusion**						
								**Day 1**						
Assessment			**20 ± 10 min**	**1h ± 15 min**	**3h ± 30 min**	**6h ± 30 min (before stop infusion)**		**7h ± 15 min**	**8h ± 30 min**	**10h ± 30 min**	**Day 2 (24h)**	**Day 3 (48 h)**	**Day 4 (72 h)**	
Screening/Administrative														
Informed consent form (ICF)[a]	X													
Inclusion/exclusion criteria	X													
Demographic data	X													
Medical and surgical history	X													
Glasgow Coma Scale	X													
APACHE IV	X													
Urine pregnancy test	X													
Safety, tolerability and efficacy evaluations														
Vital signs	X	X			X	X					X	X	X	
Height and weight	X													
12-lead ECG		X			X	X					X			
Respiratory & Fluid Support	X					X					X	X	X	
Bleeding, thrombosis and local infusion side reaction assessment	X					X					X	X	X	
SOFA (Sequential (Sepsis-related) Organ Failure Assessment)	X					X					X	X	X	
Clinical Outcomes														X
Laboratory sampling														
Hematology	X	X			X	X					X	X	X	
Coagulation	X	X			X	X			X	X	X	X	X	
Biochemistry	X	X			X	X					X	X	X	

Phase	**Screening / Baseline**	**Pre-dose**	**Continuous IV infusion with M6229**					**Post IV infusion**						**Long term follow-up (Day 30) or Early Withdrawal**
			**Time after Start of Infusion**					**Time after Start of Infusion**						
Assessment			**20 ± 10 min**	**1h ± 15 min**	**3h ± 30 min**	**6h ± 30 min (before stop infusion)**		**Day 1**			**Day 2 (24h)**	**Day 3 (48 h)**	**Day 4 (72 h)**	
								**7h ± 15 min**	**8h ± 30 min**	**10h ± 30 min**				
Histone monitoring		X				X				X	X	X	X	
Biomarkers		X				X				X	X			
Study drug administration														
IV administration of M6229			Continuous IV infusion											
Pharmacokinetics														
PK blood sampling for M6229		X	X	X	X	X		X	X	X	X			
PK urine collection for M6229		X				X				X	X			
Ongoing Subject Review														
Adverse events (AEs)	Continuously throughout the study until last treatment follow up													
Concomitant medication														

Note: whenever a patient was discharged from the ICU, the sampling assessment stopped.
