# Supplementary material for: A phase I trial evaluating the safety, tolerability, pharmacokinetics and pharmacodynamics of intravenously administered low-anticoagulant heparin (M6229) in critically ill sepsis patients
Source: Intensive Care Med Exp. 2025 Aug 18;13:84. doi: 10.1186/s40635-025-00790-4 (PMC12360993; doi:10.1186/s40635-025-00790-4)
Supplement: Supplementary file 3 — Supplementary Material 3. [file 40635_2025_790_MOESM3_ESM.pdf]

Appendix III – Adverse events definitions

Adverse events (AEs)

Adverse events were defined as any undesirable experience occurring to a subject during the study, whether or not considered related to the investigational product. The Investigator or qualified designee were responsible for assessing AEs. All adverse events reported spontaneously by the subject or observed by the investigator or his staff were recorded from the time of signing informed consent until Day 4. If the patient experienced an AE after signing consent, but did not receive study drug dosing, it was only be reported as an AE if the Investigator believed that the event may have been caused by a protocol-required procedure. AEs were described in acceptable medical terminology using a single reached diagnosis (versus signs and symptoms of the reached diagnosis).

In the ICU, various findings (clinical or laboratory tests) frequently change ("waxing and waning") or are considered usual and expected in the setting of a severely ill patient with sepsis. Therefore, these findings did not necessarily constitute a reportable AE unless they required significant intervention, lead to discontinuation of the study drug, or were considered to be of concern in the investigator's clinical judgement. Table 5 contains a list of defined conditions occurring in the ICU setting which did not have to be reported as AEs.

For specific findings in the below list, if the defined conditions for "non-AE findings" is not fulfilled, the occurring AE had to be recorded and reported.

Table A1 - Clinical Laboratory Tests

**Findings**	**Non-AE findings only if condition is met**
Obstipation	
Flatulence	
Pressure ulcers	
Hypercapnia	
Chills	Occurrence before IMP infusion or > 2h after IMP infusion

Loss of voice	
Gastric retention	
Fatigue	
Respiratory Acidosis	
Hypoxia	
Loss of muscle strength / critical illness myopathy	
Sore throat	
Hypotension	Mean Arterial Pressure < 65 mmHg for ≤ 1h or when norepinephrine is administered
Hypertension	MAP > 100 mmHg for ≤ 1 h and no vasopressor is administered
Bradycardia	Heart rate < 50 for ≤ 1 h OR when treated
Tachycardia	Heart rate > 130 for ≤ 1 h OR when treated
	Non-symptomatic
	Non-symptomatic
Hyperchloremia	
Hypokalemia	Non-symptomatic OR ≥ 3.0 mmol/L
Hyperkalemia	Non-symptomatic OR ≤ 6.5 mmol/L
Hypomagnesemia	Non-symptomatic OR ≥ 0.4 mmol/L
Hypermagnesemia	Non-symptomatic
Hyponatremia	Non-symptomatic OR ≥ 125 mmol/L
Hypernatremia	Non-symptomatic OR ≤ 155 mmol/L
Hypophosphatemia	Non-symptomatic OR ≥ 0.63 mmol/L
Hyperphosphatemia	Non-symptomatic OR ≤ 1.5 mmol/L

AEs were followed for 30 days after the end of infusion, or until resolution, whichever comes first.

The severity of an AE provides a qualitative assessment of the extent or intensity of an AE, as determined by the Investigator. The severity does not always reflect the clinical seriousness of the event, only the degree or extent of the affliction or occurrence (e.g. severe nausea, mild seizure).

The severity grade was evaluated and recorded according to the grading described in the NCI Common Terminology Criteria for Adverse Events (CTCAE), version 5.0 (NCI 2017). The severity grade refers to the severity of the AE. The CTCAE displays Grades 1 through 5 with unique clinical descriptions of severity for each AE based on the following guideline:

Grade 1: Mild; asymptomatic or mild symptoms; clinical or diagnostic observations only; intervention not indicated

Grade 2: Moderate; minimal, local or noninvasive intervention indicated; limiting age-appropriate instrumental activities of daily living

Grade 3: Severe or medically significant but not immediately life-threatening; hospitalization or prolongation of hospitalization indicated; disabling; limiting self-care activities of daily living

Grade 4: Life-threatening consequences; urgent intervention indicated

Grade 5: Death related to AE

Serious adverse events (SAEs)

A serious adverse event is any untoward medical occurrence or effect that

- results in death;
- is life threatening (at the time of the event);
- requires hospitalisation or prolongation of existing inpatients' hospitalisation;
- results in persistent or significant disability or incapacity;
- is a congenital anomaly or birth defect; or
- any other important medical event that did not result in any of the outcomes listed above due to medical or surgical intervention but could have been based upon appropriate judgement by the investigator.

All SAEs were reported by the investigator to the Sponsor within 24 hours of knowledge of their occurrence, independent of the circumstances or suspected cause. The SAE included a causality assessment. Simultaneously, other relevant information about the SAE were recorded. For all SAEs occurring during the study, the SAE was followed until the SAE was resolved, until the condition stabilized (in the case of persistent impairments), or the patient died.

The Sponsor reported all SAEs, regardless of their relationship to M6229, through the web portal ToetsingOnline to the accredited METC that approved the protocol. SAEs that resulted in death or are life threatening had to be reported within 7 days of first knowledge followed by a period of maximum of 8 days to complete the initial preliminary report. All other SAEs had to be reported within a period of maximum 15 days after the sponsor has first knowledge of the serious adverse events.

If the patient experienced a SAE after signing consent, and before study drug dosing, it was only reported as a SAE if the Investigator believed that the event may have been caused by a protocol-required procedure. Other event information to be collected inlcuded (but was not limited to): onset date, assessment of severity (CTCAE grading), relationship to the IMP (i.e., causality assessment), action taken, outcome, and date of resolution/ stabilization of the event.

Relationship to study drug

The Investigator determined whether there was a reasonable relationship between the occurrence of the AE and exposure to the study drug. Medical judgment should be used to determine the relationship, considering all relevant factors, including the pattern of reaction, temporal relationships, positive de-challenge or re-challenge relevant medical history, and confounding factors such as co-medication or concurrent diseases. The expression “reasonable causal relationship” is meant to convey in general that there are facts or arguments to suggest a causal relationship (ICH E2A).

The investigator will use medical consideration to determine the relatedness of an AE with the study drug based on the following definitions:

Unrelated

This category applies to AEs that are due to extraneous causes (disease, concomitant medication, environment, etc.) and are not related to the administration of study drug.

Unlikely Related

This category applies to AEs that are unlikely related to the administration of the study drug. The relationship of an AE to the study drug can be considered unlikely related if (must have first two criteria listed below):

- The AE does not follow a reasonable temporal sequence from administration of the drug
- The AE could readily have been a result of the patient's clinical state or other underlying medical condition, environmental or toxic factors, or other modes of therapy administered to the patient
- The AE does not follow a known response pattern to the suspected drug
- The AE does not reappear or worsen when the study drug is re-administered.

Possibly Related

This category applies to AEs that are unlikely to be related to the administration of the study drug, but the possibility cannot be ruled-out with certainty. The relationship of an AE to the study drug can be considered possibly related if (must have first two criteria listed below):

- The AE follows a reasonable temporal sequence from administration of the study drug
- The AE could readily have been a result of the patient's clinical state, environmental or toxic factors, or other modes of therapy administered to the patient
- The AE follows a known response pattern to the suspected study drug

Probably Related

This category applies to AEs that are considered with a high degree of certainty to be related to the administration of the study drug. The relationship of an AE to the study drug can be considered probably related if (must have first three criteria listed below):

- The AE follows a reasonable temporal sequence from administration of the study drug
- The AE could not be reasonably explained by the known characteristics of the patient's clinical state, environmental or toxic factors, or other modes of therapy administered to the patient
- The AE disappears or decreases upon cessation of study drug or reduction in dose
- The AE follows a known response pattern to the suspected study drug

Definitely Related

This category applies to AEs that are determined with certainty to be related to the administration of the study drug. The relationship of an AE to the study drug can be considered definitely related if (must have first three criteria listed below):

- The AE follows a reasonable temporal sequence from administration of the study drug or study drug levels have been established in body fluids or tissues
- The AE could not be reasonably explained by the known characteristics of the patient's clinical state, environmental or toxic factors, or other modes of therapy administered to the patient
- The AE disappears or decreases upon cessation of study drug or reduction in dose and, if applicable, appears upon re-challenge
- The AE follows a known response pattern to the suspected study drug
- There are exceptions when an AE does not disappear upon discontinuation of the study drug.

Follow-up of adverse events

All AEs will be followed until they have abated, or until a stable situation has been reached. Depending on the event, follow up may require additional tests or medical procedures as indicated, and/or referral to the general physician or a medical specialist. SAEs needed to be reported until the end of study within the Netherlands.
