# Supplementary material for: A phase I trial evaluating the safety, tolerability, pharmacokinetics and pharmacodynamics of intravenously administered low-anticoagulant heparin (M6229) in critically ill sepsis patients
Source: Intensive Care Med Exp. 2025 Aug 18;13:84. doi: 10.1186/s40635-025-00790-4 (PMC12360993; doi:10.1186/s40635-025-00790-4)
Supplement: Supplementary file 4 — Supplementary Material 4. [file 40635_2025_790_MOESM4_ESM.pdf]

Appendix IV – Adverse Events

Summary of adverse events per system organ class and dose level

System Organ Class (SOC)	Adverse Event	Severity	Relationship	Dose level 1	Dose level 2	Dose level 3
Blood and lymphatic system disorders	Anemia	Moderate	Not related	0	0	1 (AMC009)
Cardiac disorders	Atrial fibrillation	Moderate	Not related	0	0	2 (AMC009, AMC011)
Infections and infestations	Pulmonary aspergillus fumigatus	Moderate	Not related	1 (AMC002)	0	0
	HSV1	Mild	Not related	0	0	1 (AMC008)
Investigations	Increased ALAT	Mild	Not related	1 (AMC003)	0	1 (AMC009)
	Increased ASAT	Mild	Not related	0	0	2 (AMC007, AMC009)
	Increased ASAT/ALAT	Moderate	Unlikely related	0	0	1 (AMC011)
	Increased CK	Moderate	Unlikely related	0	0	1 (AMC011)
	Increased D-dimer	Mild	Possibly related	0	0	1 (AMC009)
	Increased Gamma-GT	Mild	Not related	1 (AMC003)	0	2 (AMC007, AMC010)
	Increased LDH	Moderate	Unlikely related	0	0	1 (AMC011)
	Increased QTc	Mild	Not related	0	1 (AMC004)	0
	Increased QTc	Mild	Probably related	0	0	1 (AMC010)
	Increased QTc	Mild	Unlikely related	0	1 (AMC004)	1 (AMC011)
	Increased alkaline phosphatase	Mild	Not related	1 (AMC003)	0	2 (AMC007, AMC010)
	Increased alkaline phosphatase	Mild	Unlikely related	0	0	1 (AMC011)
	Increased alkaline phosphatase	Mild	Not related	0	0	1 (AMC010)

**System Organ Class (SOC)**	**Adverse Event**	**Severity**	**Relationship**	**Dose level 1**	**Dose level 2**	**Dose level 3**
	Increased creatinine	Mild	Not related	0	0	2 (AMC007, AMC009)
	Increased creatinine	Moderate	Not related	1 (AMC002)	0	0
	Increased creatinine	Moderate	Unlikely related	0	0	1 (AMC011)
	Increased lipase	Mild	Not related	0	1 (AMC004)	1 (AMC009)
	Increased total cholesterol	Mild	Not related	0	1 (AMC005)	0
	Thrombocytopenia	Mild	Possibly related	0	0	1 (AMC009)
	Thrombocytopenia	Moderate	Possibly related	0	0	2 (AMC007, AMC011)
Metabolism and nutrition disorders	Hypertriglyceridemia	Mild	Not related	0	0	2 (AMC008, AMC010)
	Hypertriglyceridemia	Moderate	Unlikely related	0	0	1 (AMC006)
	Hypophosphatemia (<0.63 mmol/L)	Mild	Not related	0	1 (AMC004)	2 (AMC007, AMC008)
Nervous system disorders	Altered consciousness and twitching	Moderate	Not related	0	0	1 (AMC008)
	Seizures	Moderate	Not related	0	0	1 (AMC011)
Psychiatric disorders	Delirium	Moderate	Unlikely related	0	1 (AMC004)	0
Respiratory, thoracic and mediastinal disorders	Respiratory insufficiency	Life-threatening	Not related	0	1 (AMC004)	1 (AMC006)
Vascular disorders	Hemodynamic instability	Moderate	Not related	0	0	1 (AMC010)
	Hypertension	Moderate	Not related	1 (AMC003)	0	0
Injury, poisoning and procedural complications	Bloody leakage pleural drain	Mild	Possibly related	0	0	1 (AMC010)

Overview of all adverse events reported

Dose Level	Patient	Adverse Event	Severity	Relationship to M6229	Action take with regards to M6229	Outcome of the adverse event	Serious adverse event	System Organ Class
**Dose level 1**	*AMC002*	Aspergillus fumigatus in lung	Moderate	Not related	None	Unknown	No	Infections and infestations
		Increased creatinine	Moderate	Not related	None	Resolved	No	Investigations
	AMC003	Hypertension	Moderate	Not related	None	Resolved	No	Vascular disorders
		Increased ALAT	Mild	Not related	None	Unknown	No	Investigations
		Increased Gamma-GT	Mild	Not related	None	Unknown	No	Investigations
		Increased alkaline phosphatase	Mild	Not related	None	Resolved	No	Investigations
**Dose level 2**	*AMC004*	Delirium	Moderate	Unlikely related	None	Resolved	No	Psychiatric disorders
		Hypophosphatemia (<0.63 mmol/L)	Mild	Not related	None	Resolved	No	Metabolism and nutrition disorders
		Hypophosphatemia (<0.63 mmol/L)	Mild	Not related	None	Resolved	No	Metabolism and nutrition disorders
		Increased QTc	Mild	Not related	None	Resolved	No	Investigations
		Increased QTc	Mild	Unlikely related	None	Resolved	No	Investigations
		Increased lipase	Mild	Not related	None	Unknown	No	Investigations
		Respiratory insufficiency	Life-threatening	Not related	None	Resolved	Yes	Respiratory, thoracic and mediastinal disorders
	AMC005	Increased total cholesterol	Mild	Not related	None	Unknown	No	Investigations
**Dose level 3**	*AMC006*	Hypertriglyceridemia	Moderate	Unlikely related	None	Unknown	No	Metabolism and nutrition disorders
		Respiratory insufficiency	Life-threatening	Not related	None	Resolved	Yes	Respiratory, thoracic and mediastinal disorders

Dose Level	Patient	Adverse Event	Severity	Relationship to M6229	Action take with regards to M6229	Outcome of the adverse event	Serious adverse event	System Organ Class
	AMC007	Hypophosphatemia (<0.63 mmol/L)	Mild	Not related	None	Resolved	No	Metabolism and nutrition disorders
		Increased ASAT	Mild	Not related	None	Unknown	No	Investigations
		Increased Gamma-GT	Mild	Not related	None	Unknown	No	Investigations
		Increased alkaline phosphatase	Mild	Not related	None	Unknown	No	Investigations
		Increased creatinine	Mild	Not related	None	Resolved	No	Investigations
		Thrombocytopenia	Moderate	Possibly related	None	Resolved	No	Investigations
	AMC008	Altered consciousness and twitching	Moderate	Not related	None	Resolved	No	Nervous system disorders
		HSV1	Mild	Not related	None	Unknown	No	Infections and infestations
		Hypertriglyceridemia	Mild	Not related	None	Unknown	No	Metabolism and nutrition disorders
		Hypophosphatemia (<0.63 mmol/L)	Mild	Not related	None	Resolved	No	Metabolism and nutrition disorders
	AMC009	Anemia	Moderate	Not related	None	Resolved	No	Blood and lymphatic system disorders
		Atrial fibrillation	Moderate	Not related	None	Resolved	No	Cardiac disorders
		Increased ALAT	Mild	Not related	None	Unknown	No	Investigations
		Increased ASAT	Mild	Not related	None	Resolved	No	Investigations
		Increased D-dimer	Mild	Possibly related	None	Unknown	No	Investigations
		Increased creatinine	Mild	Not related	None	Resolved	No	Investigations
		Increased lipase	Mild	Not related	None	Unknown	No	Investigations

Dose Level	Patient	Adverse Event	Severity	Relationship to M6229	Action take with regards to M6229	Outcome of the adverse event	Serious adverse event	System Organ Class
		Thrombocytopenia	Mild	Possibly related	None	Resolved	No	Investigations
	AMC010	Bloody leakage pleural drain	Mild	Possibly related	None	Resolved	No	Injury, poisoning and procedural complications
		Hemodynamic instability	Moderate	Not related	None	Resolved	No	Vascular disorders
		Hypertriglyceridemia	Mild	Not related	None	Unknown	No	Metabolism and nutrition disorders
		Increased Gamma-GT	Mild	Not related	None	Unknown	No	Investigations
		Increased QTc	Mild	Probably related	None	Resolved	No	Investigations
		Increased alkaline phosphatase	Mild	Not related	None	Unknown	No	Investigations
		Increased alkaline phosphatase	Mild	Not related	None	Unknown	No	Investigations
		Increased alkaline phosphatase	Mild	Not related	None	Resolved	No	Investigations
	AMC011	Atrial fibrillation	Moderate	Not related	None	Resolved	No	Cardiac disorders
		Increased ASAT/ALAT	Moderate	Unlikely related	None	Death	No	Investigations
		Increased CK	Moderate	Unlikely related	None	Death	No	Investigations
		Increased LDH	Moderate	Unlikely related	None	Death	No	Investigations
		Increased QTc	Mild	Unlikely related	None	Resolved	No	Investigations
		Increased alkaline phosphatase	Mild	Unlikely related	None	Death	No	Investigations
		Increased creatinine	Moderate	Unlikely related	None	Death	No	Investigations

Dose Level	Patient	Adverse Event	Severity	Relationship to M6229	Action take with regards to M6229	Outcome of the adverse event	Serious adverse event	System Organ Class
		Seizures	Moderate	Not related	None	Resolved	No	Nervous system disorders
		Thrombocytopenia	Moderate	Possibly related	None	Resolved	No	Investigations
