# Supplementary figures and images for: A phase I trial evaluating the safety, tolerability, pharmacokinetics and pharmacodynamics of intravenously administered low-anticoagulant heparin (M6229) in critically ill sepsis patients
Source: Intensive Care Med Exp. 2025 Aug 18;13:84. doi: 10.1186/s40635-025-00790-4 (PMC12360993; doi:10.1186/s40635-025-00790-4)

## Appendix V – Vital Signs

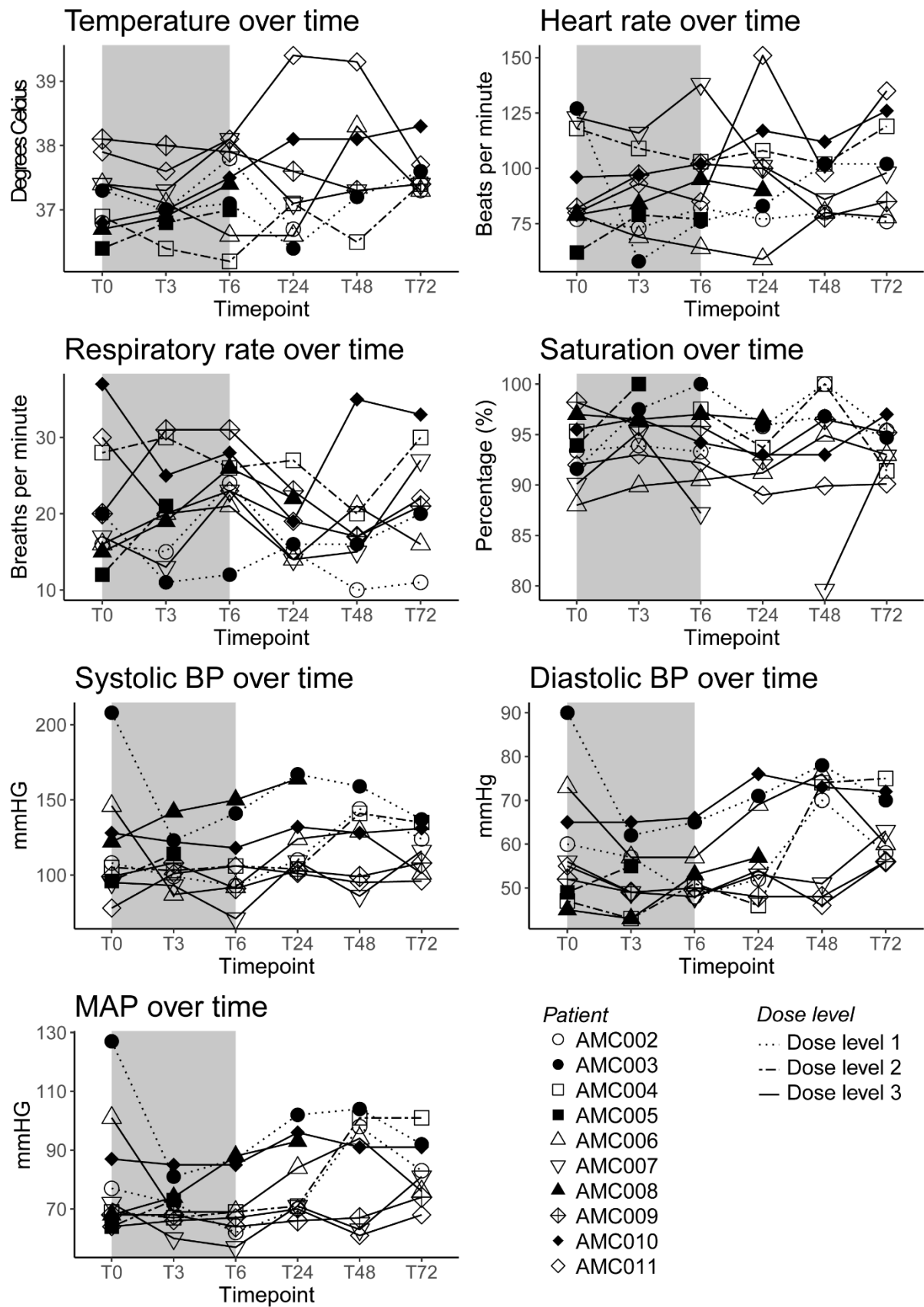

Supplement: Supplementary file 5 — Supplementary Material 5. [file 40635_2025_790_MOESM5_ESM.pdf]

## Appendix VII – Coagulation

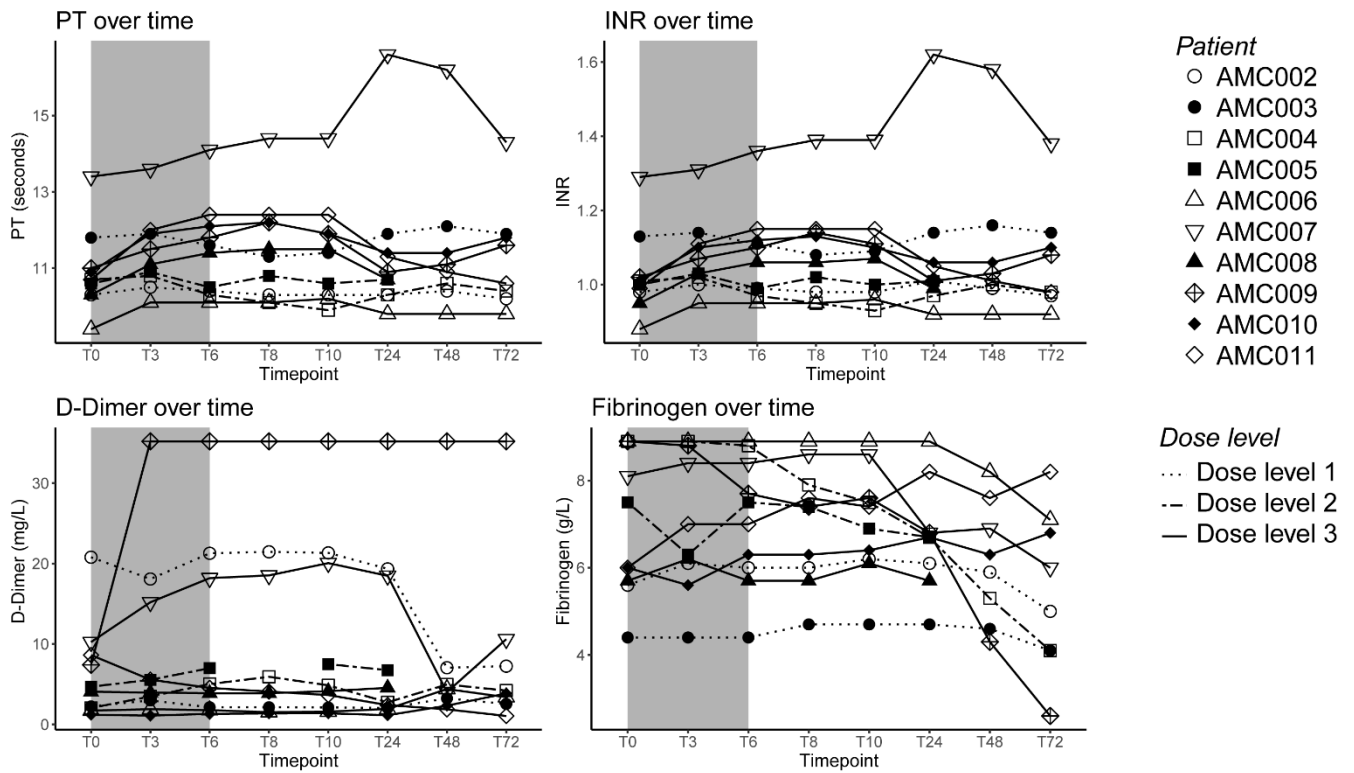

Supplement: Supplementary file 7 — Supplementary Material 7. [file 40635_2025_790_MOESM7_ESM.pdf]

# Appendix VIII - Biochemistry

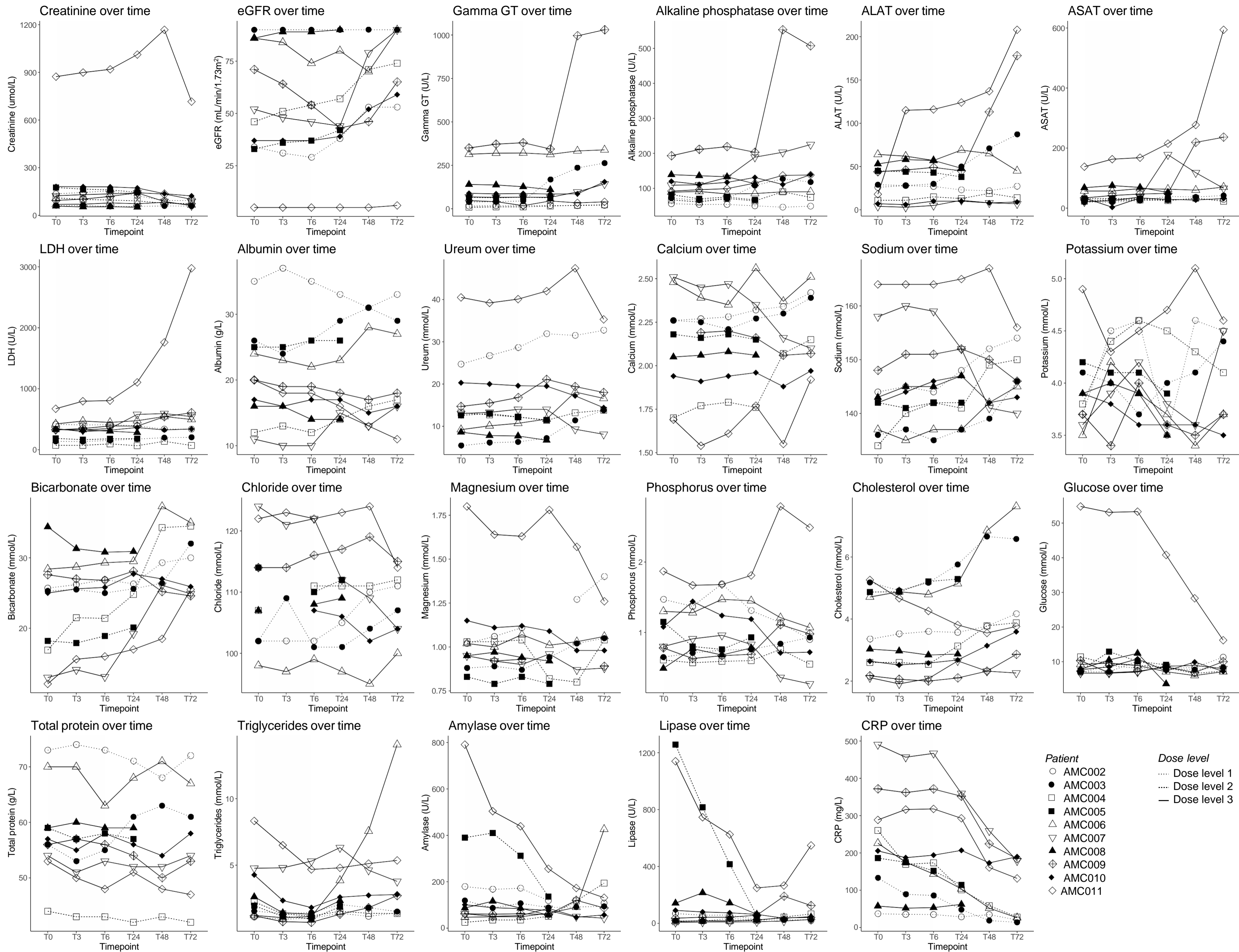

Supplement: Supplementary file 8 — Supplementary Material 8. [file 40635_2025_790_MOESM8_ESM.pdf]
