# Supplementary material for: A phase I trial evaluating the safety, tolerability, pharmacokinetics and pharmacodynamics of intravenously administered low-anticoagulant heparin (M6229) in critically ill sepsis patients
Source: Intensive Care Med Exp. 2025 Aug 18;13:84. doi: 10.1186/s40635-025-00790-4 (PMC12360993; doi:10.1186/s40635-025-00790-4)
Supplement: Supplementary file 6 — Supplementary Material 6. [file 40635_2025_790_MOESM6_ESM.pdf]

Appendix VI – Hematology

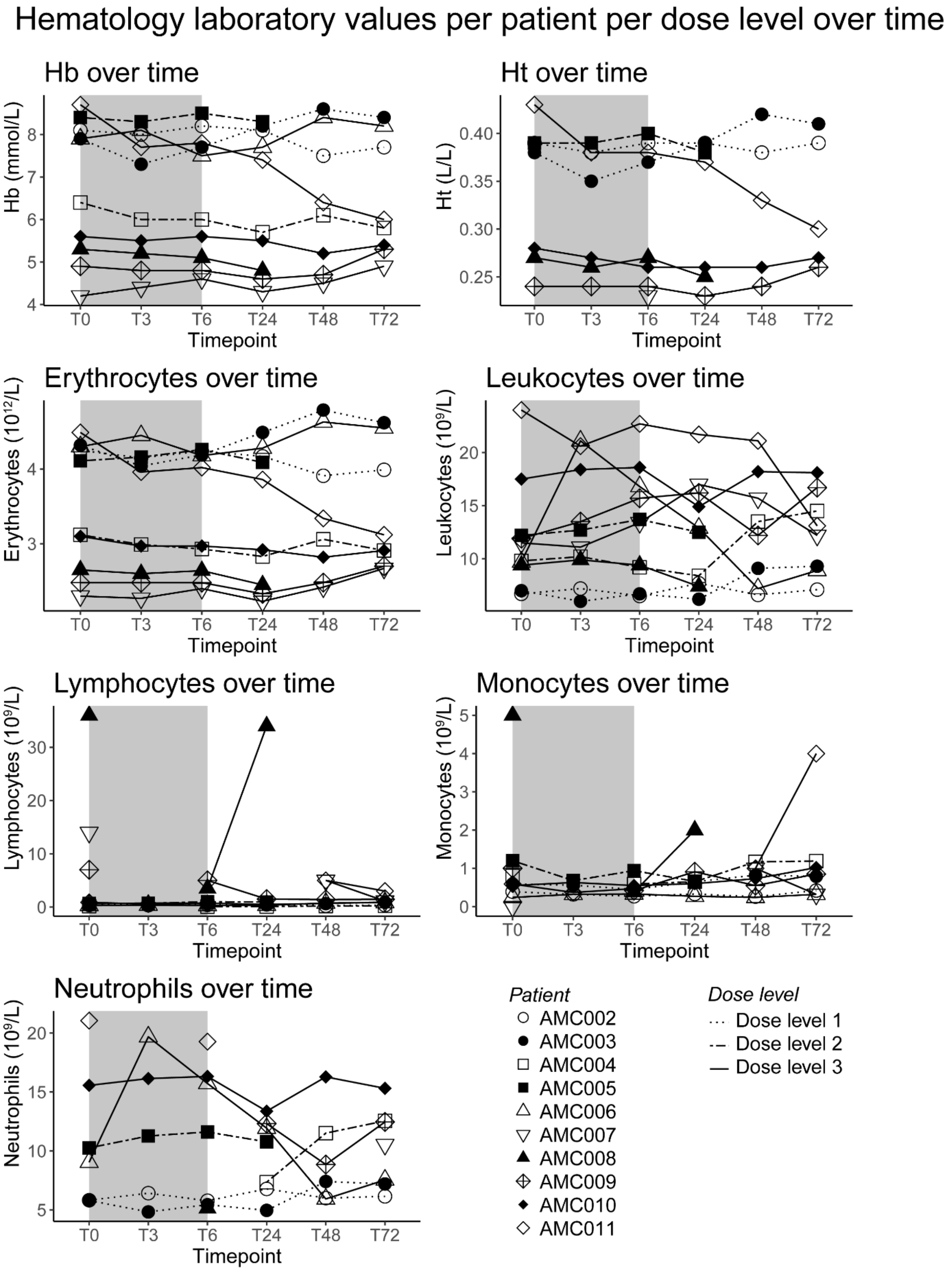

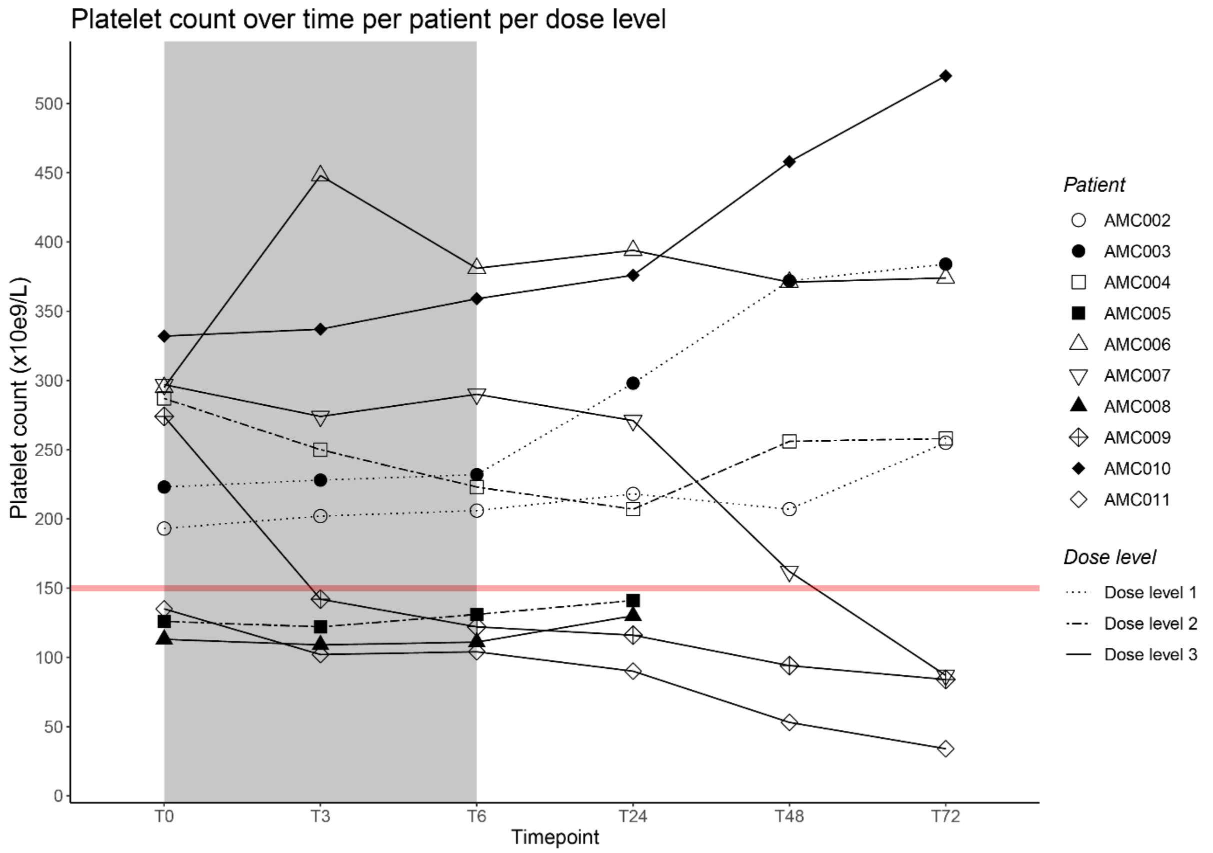

Platelet count over time per patient per dose level
Platelet count (x10e9/L)
500
450
400
350
300
250
200
150
100
50
0
T0
T3
T6
T24
T48
T72
Timepoint
Patient
AMC002
AMC003
AMC004
AMC005
AMC006
AMC007
AMC008
AMC009
AMC010
AMC011
Dose level
Dose level 1
Dose level 2
Dose level 3
