# Supplementary material for: A phase I trial evaluating the safety, tolerability, pharmacokinetics and pharmacodynamics of intravenously administered low-anticoagulant heparin (M6229) in critically ill sepsis patients
Source: Intensive Care Med Exp. 2025 Aug 18;13:84. doi: 10.1186/s40635-025-00790-4 (PMC12360993; doi:10.1186/s40635-025-00790-4)
Supplement: Supplementary file 9 — Supplementary Material 9. [file 40635_2025_790_MOESM9_ESM.pdf]

Appendix IX – ECG Measurements

Patient	Timepoint	Heart rate (beats per minute)	PR interval (ms)	QRS interval (ms)	QT interval (ms)
AMC002	T0	70	143	107	434
	T3	73	136	109	414
	T6	81	130	100	384
	T24	74	139	109	410
AMC003	T0	66	134	96	410
	T3	60	133	96	432
	T6	76	130	96	314
	T24	79	129	94	366
AMC004	T0	122	163	85	327
	T3	106	180	84	361
	T6	100	186	91	371
	T24	115	165	86	361
AMC005	T0	66	174	94	441
	T6	84	166	91	404
AMC006	T0	83	150	105	373
	T6	63	143	108	405
	T24	56	145	110	471
AMC007	T0	108	158	116	367
	T3	115	152	111	311
	T6	136	158	112	332
	T24	110	NA	111	315
AMC008	T0	79	149	82	390
	T3	79	149	82	390
	T6	85	143	91	400
	T24	91	145	89	374
AMC009	T0	99	152	86	334
	T3	102	153	91	340
	T6	103	151	85	333
	T24	87	130	86	378
AMC010	T0	91	120	92	401
	T3	96	137	90	396
	T6	103	134	86	421
	T24	86	128	94	419
AMC011	T0	90	140	103	388

	T3	84	149	90	407
	T6	83	142	94	408
	T24	91	146	97	390
