# Supplementary material for: A phase I trial evaluating the safety, tolerability, pharmacokinetics and pharmacodynamics of intravenously administered low-anticoagulant heparin (M6229) in critically ill sepsis patients
Source: Intensive Care Med Exp. 2025 Aug 18;13:84. doi: 10.1186/s40635-025-00790-4 (PMC12360993; doi:10.1186/s40635-025-00790-4)
Supplement: Supplementary file 10 — Supplementary Material 10. [file 40635_2025_790_MOESM10_ESM.pdf]

Appendix X - renal excretion of M6229 after administration of M6229 by a 6-hour intravenous infusion to ICU admitted patients with sepsis in HistoSeps study

Assessment of M6229 excreted in urine was an exploratory outcome in the study plan. Moreover, urine samples and urine volumes were not taken for all patients at all time points, and hence, excretion can only be determined for 5 of 10 patients. Given the exploratory nature and limited amount of data, only a preliminary data analysis was conducted.

In HistoSeps, patients admitted to the intensive care unit with sepsis, received M6229 by a 6-hour intravenous (IV) infusion according to the following nominal dose groups: 0.15 mg/kg/hr (patients AMC002 and 3), 0.45 mg/kg/hr (patients AMC004 and 5), 0.90 mg/kg/hr (patients AMC006, 7, 8, 9, 10 and 11). The actual total dose per patient is presented in the table below.

Urine was collected from start of the infusion to the end-of-infusion (time 0 - 6 hours), during 4 hours after end- of-infusion (6 – 10 hours) and between 10 and 24 hours (10-24 hours). Volume of the urine was determined and a sample taken for measurement. The M6229 concentration in urine was measured by a qualified assay based on ligand binding to HepRed dye (Ardena R23383). In addition, urine samples taken just before administration were measured.

M6229 was detected in urine collected from 0 to 6 hours in nearly all patients. Two patients did not have M6229 in urine at any time point: AMC007 who also did not have plasma PK profile congruent to IV infusion; and AMC011 who had an extremely low glomerular filration rate indicative of renal failure (5 ml/min). In samples taken before administration, only patient AMC004 showed a positive signal in urine, potentially due to presence of endogenous glycoaminoglycans or previous treatment with LMWH/heparin.

Overall, only a minor amount of M6229 was excreted in urine of sepsis patients ranging between 0.3 and 2.9% of the administerd dose (mean 1.9%; median 2.7%). This excretion was already reached in the first 10 hours after start of the 6-hour infusion. The estimated renal clearance is low compared to the total clearance as determined by non-compartmental analysis of plasma data (Table 1). The excretion was independent of the dose level or the glomerular filtration rate in these patients (Table 1; Figure 1). Patient AMC011 had a high plasma exposure for M6229 (Table 1), but this seems not related to the renal failure in this patient given the low percentage of urinary excretion observed in other patients.

First preliminary results for excretion of M6229 indicate that renal clearance and excretion of M6229 is very low in patients (<3% of the administered dose).

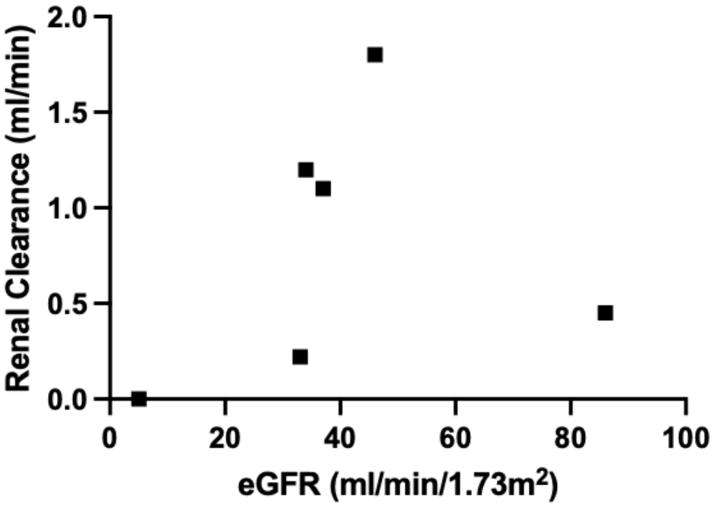

Figure 1: Renal clearance of M6229 did not correlate to the glomerular filtration rate. Renal clearance was low compared to the total clearance of M6229 in these patients (median: 47 ml/min (range 37-78 ml/min; excluding patient AMC011).

Table 1: Urinary excretion of M6229 during infusion in patients

Patient	Dose (mg)	Cu,0h (ug/ml)	Cu,0-6h (ug/ml)	Vu,0-6h (ml)	Ae,0-6h (mg)	Cu,6-10h (ug/ml)	Vu,6-10h (ml)	Ae,6-10h (mg)	Cu,10-24h (ml)	Vu,10-24h (ml)	Ae,10-24h (mg)
**AMC002**	**66**	<0.75	1.87	810	1.5	0.967	285	0.3	<0.750	--	0
**AMC003**	**72**	<0.75	8.53	--	--	<0.75	--	0	<0.750	--	0
**AMC004**	**135**	1.90	4.28	440	1.9	4.13	500	2.1	<0.750	2000	0
**AMC005**	**230**	<0.75	1.37	270	0.4	0.967	300	0.3	--	--	--
**AMC006**	**420**	<0.75	9.84	335	3.3	8.79	--	--	--	--	--
**AMC007**	**330**	<0.75	<0.75	--	0	<0.75	--	0	0	--	0
**AMC008**	**450**	<0.75	8.65	400	3.5	6.43	50	0.3	--	--	--
**AMC009**	**388.8**	<0.75	10.0	--	--	7.31	--	--	<0.750	--	0
**AMC010**	**430**	<0.75	10.6	700	7.4	8.04	600	4.8	--	--	--
**AMC011**	**394**	<0.75	<0.75	15	0	<0.75	--	0	<0.750	100	0

Patient	Dose (mg)	Ae,0-24h (mg)	% of dose excreted (0-24h)	AUCinf (ug.h/ml)	Cl renal (ml/min)	eGFR (ml/min/1.7m^2)	Cl total (ml/min)
**AMC002**	**66**	1.8	2.7%	25.8	1.2	34	42.7
**AMC003**	**72**	--	--	8.21	--	90	
**AMC004**	**135**	3.9	2.9%	36.7	1.8	46	61.3
**AMC005**	**230**	0.7	0.3%	49.1	0.22	33	78.0
**AMC006**	**420**	--	--	170	--	86	41.2
**AMC007**	**330**	0	0%		--	52	
**AMC008**	**450**	3.8	0.8%	140	0.45	86	53.5
**AMC009**	**388.8**	--	--	137	--	71	47.3
**AMC010**	**430**	12.2	2.8%	194	1.1	37	37.0
**AMC011**	**394**	0	0%	623	0	5	10.6

Cu concentration in urine, Vu volume urine collected, Ae amount excreted in urine, --: not reported or not possible to calcuate. Cl renal calculated by Ae0-24h/AUCinf. AMC007 did not have an AUC calculated due to the absence of a plasma PK profile. eGFR: glomerular filtration rate estimated from serum creatinine. AUC and total Clearance from PK analysis report (DGr23013).
