# Supplementary material for: A phase I trial evaluating the safety, tolerability, pharmacokinetics and pharmacodynamics of intravenously administered low-anticoagulant heparin (M6229) in critically ill sepsis patients
Source: Intensive Care Med Exp. 2025 Aug 18;13:84. doi: 10.1186/s40635-025-00790-4 (PMC12360993; doi:10.1186/s40635-025-00790-4)
Supplement: Supplementary file 11 — Supplementary Material 11. [file 40635_2025_790_MOESM11_ESM.pdf]

Appendix XI – Histone Cleavage

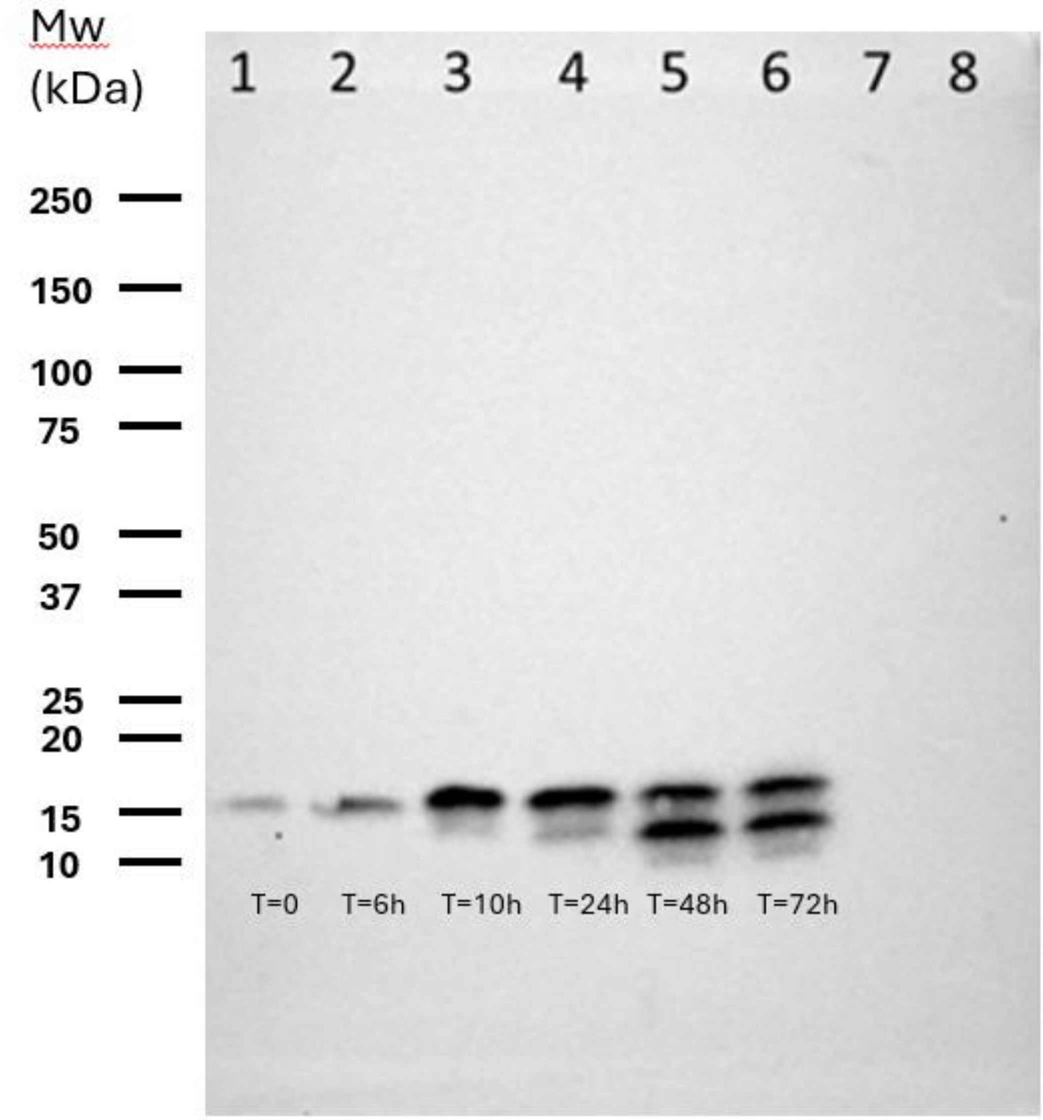

Analysis of histone presence in patient plasma by Western blotting. Samples were prepared and SDS-PAGE and subsequent Western blotting were performed as described in Methods. Visualization of human histone H3 was done by using an anti-histone H3 antibody. The blot shows 6 lanes containing the samples collected from patient AMC009 at different time points. Uncleaved histone H3 was observed at all time points sampled whereas cleaved histone H3 could increasingly be observed for the samples taken at T=10, 24, 48 and 72h.
